# Sporadic Creutzfeldt–Jakob disease subtype-specific alterations of the brain proteome: Impact on Rab3a recycling

**DOI:** 10.1002/pmic.201200201

**Published:** 2012-12-12

**Authors:** Joanna Gawinecka, Franco Cardone, Abdul R Asif, Angela De Pascalis, Wiebke M Wemheuer, Walter J Schulz-Schaeffer, Maurizio Pocchiari, Inga Zerr

**Affiliations:** 1 National Reference Center for TSE Surveillance, Medical Center Georg-August UniversityGoettingen, Germany; 2 Department of Cell Biology and Neurosciences, Istituto Superiore di SanitàRome, Italy; 3 Department of Clinical Chemistry, Medical Center Georg-August UniversityGoettingen, Germany; 4 Department of Neuropathology, Medical Center Georg-August UniversityGoettingen, Germany

**Keywords:** Biomedicine, 2D-DIGE, Prion, Proteome, Rab3a, Sporadic Creutzfeldt–Jakob disease

## Abstract

Sporadic Creutzfeldt–Jakob disease (sCJD) is characterized by wide clinical and pathological variability, which is mainly influenced by the conformation of the misfolded prion protein, and by the methionine and valine polymorphism at codon 129 of the prion protein gene. This heterogeneity likely implies differences in the molecular cascade that leads to the development of certain disease phenotypes. In this study, we investigated the proteome of the frontal cortex of patients with the two most common sCJD subtypes (MM1 and VV2) using 2D-DIGE and MS. Analysis of 2D maps revealed that 46 proteins are differentially expressed in the sCJD. Common differential expression was detected for seven proteins, four showed opposite direction of differential expression, and the remaining ones displayed subtype-specific alteration. The highest number of differentially expressed proteins was associated with signal transduction and neuronal activity. Moreover, functional groups of proteins involved in cell cycle and death, as well as in structure and motility included subtype-specific expressed proteins exclusively. The expression of Rab GDP dissociation inhibitor alpha, which regulates Rab3a-mediated neurotransmitter release, was affected in both sCJD subtypes that were analyzed. Therefore, we also investigated as to whether Rab3a recycling is altered. Indeed, we found an accumulation of the membrane-associated form, thus the active one, which suggests that dysfunction of the Rab3a-mediated exocytosis might be implicated in sCJD pathology.

## 1 Introduction

Sporadic Creutzfeldt–Jakob disease (sCJD), the most common form of human transmissible spongiform encephalopathies, is characterized by wide clinical and pathological variability. On the molecular level, the disease phenotype is defined by the methionine/valine polymorphism at codon 129 in the human prion protein gene (*PRNP* gene) and by the presence of two major types of pathological, protease-resistant forms of the scrapie prion protein (PrP^Sc^), leading to two different profiles in Western blot (type 1 and type 2) 1. The major subtypes of sCJD are homozygotes for methionine at codon 129 in *PRNP* gene with PrP^Sc^ type 1 (MM1), and homozygotes for valine at codon 129 in the *PRNP* gene with PrP^Sc^ type 2 (VV2) representing about 67 and 15% of all sCJD cases, respectively. The clinical and pathological characteristics of molecular disease subtypes differ markedly with respect to symptoms at onset, localization, and type of the pathological changes, as well as the PrP^Sc^ deposition pattern 1,2. This might suggest involvement of different molecular pathways in sCJD pathogenesis.

Proteomic technologies provide a unique opportunity to analyze biological processes at the protein level on a global scale. In particular, knowledge about changes in protein abundances and their modifications, caused by a disease or induced by toxic agents, can provide new insight into pathological processes and can improve our understanding of the underlying mechanisms. In turn, this knowledge can be applied to the discovery of diagnostic markers and the detection of new drug targets. Although the identification of disease-related proteome differences in human brain tissue is difficult and challenging, it is also an incomparable way to investigate disorders of the central nervous system in humans.

In this study, we analyzed brain proteome alterations in two of the most frequent sCJD subtypes: MM1 and VV2. Proteomic data indicate several commonly differentially expressed proteins in both subtypes, with the majority showing subtype-specific expression. The highest number of differentially expressed proteins was associated with signal transduction and neuronal activity. Moreover, functional groups of proteins involved in the cell cycle and death, as well as in the structure and motility exclusively included subtype-specific expressed proteins.

Two CJD-related proteins, Rab GDP dissociation inhibitor alpha (αGDI) and Hsp90 are involved in Rab3a-mediated neurotransmitter release. These three proteins were further investigated, as they may have a profound relevance for disease pathology in CJD patients.

## 2 Patients, materials, and methods

### 2.1 Patients

Samples from the frontal cortex were obtained from ten pathologically confirmed sCJD patients (five with MM1 and five with VV2 subtype) and five age-matched control cases (CON) with codon 129 polymorphism as follows: 1 × MM, 2 × MV, 2 × VV. The mean age for the MM1, VV2, and CON group was 71 ± 7, 68 ± 6, and 69 ± 7 years, respectively. Control patients were selected based on absence of the neurological disorders and were diagnosed with multi-organ dysfunction syndrome, chronic kidney dysfunction, lung cancer, or myocardial infarction. A histopathological examination of the brain tissue revealed only age-dependent changes of the brain structure and neuronal morphology (no signs of intracranial pressure, bleeding, vascular changes, or abnormalities indicating neurodegenerative processes). The sCJD patients showed typical disease duration, as well as clinical and neuropathological findings. The mean disease duration of the MM1-sCJD and VV2-sCJD subtype was 4 ± 2 and 8 ± 2 months, respectively. Moreover, all sCJD patients were demented and positive for 14–33 protein in CSF. Three MM1 and four VV2s patients displayed hyperintensity in basal ganglia detected by a T2-weighted MRI, while the presence of periodic sharp wave complexes in EEG was detected in one MM1, and in all VV2 patients. The histopathological examination revealed a CJD-characteristic lesion triad: spongiosis, neuronal loss, and astrogliosis; the immunochemistry showed PrP depositions in the brain tissue. The post mortem delay was around 24 h for all analyzed samples. Up until the autopsy procedure, corpses were stored at 4°C.

### 2.2 Sample preparation

#### 2.2.1 Preparation of brain homogenates

Prior to analysis, samples of the frontal cortex were stored at −80°C, and before homogenization were rinsed with a PBS buffer. Samples were homogenized in five volumes of a homogenization buffer containing 20 mM HEPES (pH 7.4), 0.32 M sucrose, 1 mM of sodium orthovanadate, 1 mM of EDTA, and Complete Protease Inhibitor Cocktail (Roche), using a glas-teflon homogenizer. Brain homogenates were centrifuged at 15 000 × *g* for 10 min. Supernatants were then collected, and protein concentrations were determined by the BCA assay (Sigma).

#### 2.2.2 Preparation of membrane and cytosol fractions

Brain homogenates were ultracentrifuged at 100 000 × *g* at 4°C for 1 h to separate cytosol from the membrane fraction. Membrane pellets were resuspended in the homogenization buffer. Finally, protein concentrations in both fractions were determined by the BCA assay (Sigma). The efficiency of separation was shown by a Western blot analysis directed against caveolin 1 and anti-alpha 1 sodium potassium ATPase, membrane-specific proteins, and synaptophysin, a synaptic marker (Supporting Information File 1).

### 2.3 Fluorescence 2D-DIGE

#### 2.3.1 2D-DIGE and 2D image analysis

Fifty micrograms of crude cytosol proteins (brain homogenate) were precipitated overnight with acetone-methanol (8:1; vol:vol) at −20°C and centrifuged at 16 000 × *g* for 15 min. The pellet was resuspended in 20 μL of lysis buffer containing 7 M urea, 2.5 M thiourea, 4% CHAPS, 30 mM TRIS, and 5 mM magnesium acetate and subsequently labeled with 200 pmol of CyDye (GE Healthcare) as follows: pooled samples as internal standard (IS), individual control, and sCJD samples with either Cy3 or Cy5. The dye switch between IS, control, and sCJD samples was done in order to avoid dye-to-protein preferences.

The labeling reaction was performed on ice in the dark for 30 min, terminated by adding 10 mM lysine, and was subsequently incubated for a further 10 min. Equal volumes of a lysis buffer containing additionally 130 mM DTT and 0.8% 3–10 Bio-Lyte (Bio-Rad) were added to the labeling mixture. Then the samples were mixed together, diluted up to 450 μL with a rehydration buffer composed of 7 M urea, 2.5 M thiourea, 4% CHAPS, 0.2% 3–10 Bio-Lyte and 65 mM DTT and loaded on ReadyStrip IPG nonlinear pH 3–10, 24 cm strip (Bio-Rad). After 12 h of active rehydration at 50 V, isoelectric focusing was initiated at 500 V for 1 h, followed by ramping at 1000 V for 1 h, and 5000 V for 2 h. The final focusing was carried out at 8000 V, reaching a total of 80 000 Vh (PROTEAN IEF CELL, Bio-Rad). The strips were then equilibrated twice for 20 min in a buffer containing 6 M urea, 2% SDS, 30% glycerin, and 150 mM TRIS, pH 8.8, supplemented with 2% DTT in the first, and with 2.5% IAA in the second equilibration step. SDS-PAGE was performed overnight with homogenous 12% polyacrylamide gel and constant V (80 V for 2 h and 150 V for around 16 h) using Ettan DALTsix (GE, Amersham). CyDye-labeled protein gels were scanned by two different lasers with band pass filtered emission wavelengths of 580 nm (Cy3) and 670 nm (Cy5) using Typhoon 9200 Variable Mode Imager (GE, Amersham).

Protein spot abundances within 20 brain proteome patterns (5 MM1, 5 VV2, 5 CON, and 5 IS) were analyzed using the Delta2D software (v. 3.6) (DECODON). Differences in spot abundance detected by densitometric analysis were statistically evaluated using the unpaired Student's *t-*test. A protein spot was considered as differentially expressed when the 1.7-fold change in abundance was accompanied by a *p*-value < 0.05 in the unpaired Student's *t*-test.

#### 2.3.2 Preparative 2D gels and protein identification

To identify differentially expressed protein spots, preparative 2D gels containing 350-μg protein were prepared as described above (DIGE-staining procedure was omitted). To reduce the infectivity of brain homogenates and to minimize the risk of infection for laboratory staff, samples were boiled at 95°C in 5% SDS for 10 min 3–6 before precipitation with acetone-methanol (8:1; vol:vol) at −20°C. After gel electrophoresis was terminated, gels were stained with colloidal Coomassie staining solution according to the protocol given by Candiano et al. 7. Gel plugs containing visualized proteins of interest were manually excised from gels and subjected to in-gel digestion. The detailed protocol of this procedure is given by Ramljak et al. 8. Digested peptides were dissolved in 0.1% TFA followed by injection into the Q-TOF Ultima Global mass spectrometer (Micromass, Manchester, UK) as described before 9. The data were acquired with the MassLynx (v 4.0) software on a Windows NT PC and further processed using ProteinLynx Global Server (PLGS, v 2.2, Micromass, Manchester, UK) as PKL (peak list) under the following settings: electrospray, centrioid 80% with minimum peak width 4 channel, noise reduction 10%, Savitzky-Golay, MSMS, medium deisotoping with 3% threshold, no noise reduction, and no smoothing. The peaklists were searched out using the online MASCOT algorithm against the SwissProt 2011_07 (530 264 sequences; 1 879 410 742 residues). The data obtained were retrieved against the whole database with search parameters set as follows: enzyme, trypsin; allowance of up to one missed cleavage peptide; mass tolerance ± 0.5 Da and MS/MS tolerance ± 0.5 Da; modifications of cysteine carboamidomethylation and methionine oxidation when appropriate, with auto hits allowed; only significant hits to be reported.

### 2.4 Western blotting

Ten micrograms of brain homogenate (prepared as given in Section 2.2.1), purified cytosol, or membrane proteins (prepared as given in Section 2.2.2) were separated on 12% SDS-PAGE gels and transferred to PVDF membranes. Membranes were blocked with 5% skimmed milk in PBS with 0.2% Triton X-100 (PBST) for 1 h at RT. Subsequently, membranes were incubated overnight at 4°C with one of the following primary antibodies: rabbit anti-Hsp90 (1:10 000; Abcam), mouse anti-β-actin (1:10 000; Abcam) ALDH9A1 (1:200; Abcam), rabbit anti-calmodulin (1:2000; Abcam), rabbit anti-14–3-3β (1:3000; Abcam), rabbit anti-caveolin-1 (1:150; Santa Cruz), rabbit anti-synaptophysin (1:1000; Abcam), mouse anti-alpha 1 sodium potassium ATPase (1:2000; Abcam), and mouse anti-macrophage migration inhibitory factor (1:1000; Abcam). Thereafter, membranes were washed with PBST and incubated for 1 h at RT with a corresponding horseradish peroxide-conjugated secondary antibody: goat anti-rabbit (1:10 000; Jackson Research) or goat anti-mouse (1:5000; Jackson Research). The immunoreactivity was detected after membrane immersion into ECL solution and capturing the chemiluminescent signal by ChemiDoc XRS+ System (Bio-rad).

Densitometric and statistical analysis was performed with Image Lab (Bio-rad) and Sigmaplot (Exact Graphs and Data Analysis software, Systat), respectively. The protein differential expression was considered as significant when the *p*-value was lower than 0.05 using the one-way ANOVA test with Bonferroni correction.

## 3 Results

### 3.1 Comparative brain proteome analysis using 2D-DIGE

Using 2D-DIGE technology and Delta2D's 100% spot matching approach, we detected around 670 protein spots on a proteome pattern of the human frontal cortex. Densitometric and statistical comparison of the MM1 versus CON, and VV2 versus CON revealed 60 protein spots with a significantly different expression level in the sCJD, comprising 9% of all detected spots.

The expression level of sCJD-related proteins did not show any significant differences between MM, MV, and VV genotypes in the control group, except for septin-11 and both 14–3-3 beta/alpha, and delta/zeta isoforms. It seems that the presence of methionine in the codon 129 is associated with an increased level of septin-11, and the presence of valine in the codon 129 is associated with an increased level of 14–4-3 (data not shown).

**Figure 1:**
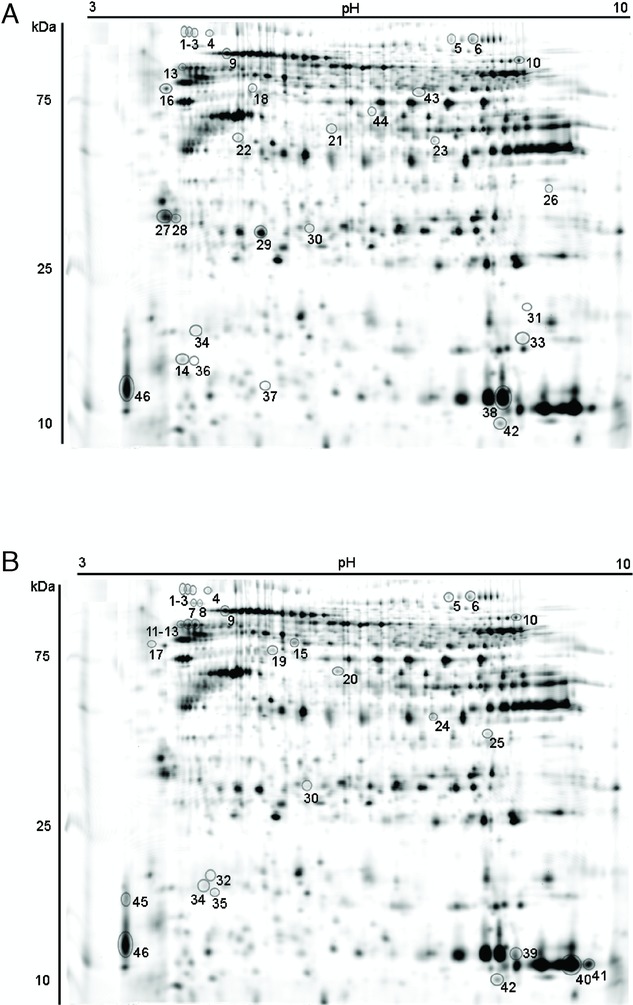
2D-DIGE maps of the crude cytosolic fraction of human frontal cortex proteome. Differentially expressed proteins in MM1 (panel A) and VV2 (panel B) sCJD subtype are marked, respectively.

Proteins were identified from 46 protein spots. From the remaining 14 protein spots, identification was not possible due to undetectable or very faint staining on colloidal Coomassie stained preparative gel. The discrepancy between the number of protein spots and proteins can be explained by the coexistence of different isoforms of the same proteins, which vary in their isoelectric point and/or molecular weight (Fig. 1).

A common differential expression in both analyzed sCJD subtypes was found for seven proteins. Among them, six showed lower expression level: one calmodulin isoform (ID 46), two αGDI isoforms (ID 12 and 13), SUMO 2/3 (ID 34a/b), one Hsp90 isoform (ID 3), and transketolase (ID 10), while heat shock cognate 71 kDa protein (ID 9) was shown to have a higher expression level. Interestingly, two Hsp90 isoforms (ID 1 and ID 4) showed the opposite direction of differential expression; their decreased level was detected in the MM1, while their level increased by one in the VV2 subtype. Higher expression levels of one mitochondrial aconitate hydratase isoform (ID 5) and macrophage migration inhibitory factor (ID 42) were found in the MM1, while their levels were lower in the VV2 subtype. The remaining proteins showed differential expression specific to a certain sCJD subtype.

**Figure 2:**
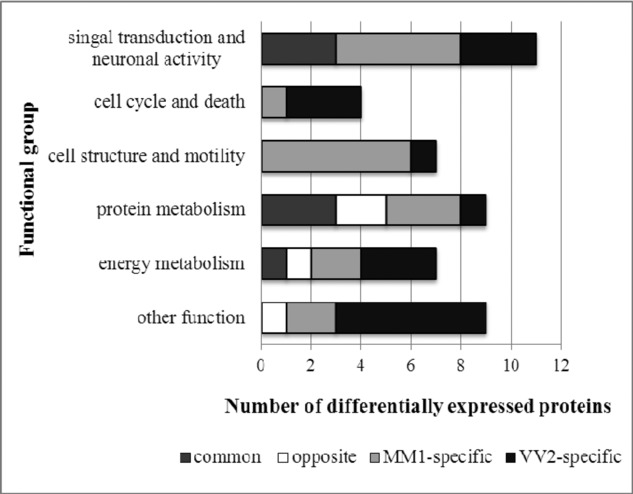
Distribution of differentially expressed proteins according to type of differential expression (common, opposite, MM1, and VV2 subtype-specific differential expression) and biological function.

Overall, differentially expressed proteins were categorized into five groups according to their biological function: signal transduction and neuronal activity; cell cycle and death; cell structure and motility; protein metabolism and energy metabolism. Several proteins could not be classified according to any of the above-mentioned groups and were subsumed under other functions. The highest number of differentially expressed proteins was found in the signal transduction and neuronal activity group. Interestingly, the cell cycle and death, as well as the cell structure and motility groups hold subtype-specific differentially expressed proteins exclusively (Table 1 and Fig. 2).

**Table 1:** List of differentially expressed proteins in the frontal cortex isolated from patients with MM1 and VV2 sCJD subtype. A protein was considered as significantly differentially expressed when the 1.7-fold change in abundance (MM1 versus CON and VV2 versus CON) was accompanied by *p*-value < 0.05 in unpaired Student's *t*-test. Additionally, proteins were clustered into six groups according to their biological function (signal transduction and neuronal activity; cell cycle and death; cell structure and motility; protein metabolism; energy metabolism and other function). ^MM1^ and ^VV2^ indicates differential expression specific for MM1 and VV2 sCJD subtype, respectively. ↓↑ indicates opposite differential expression

ID	Protein name	MM1 subtype			VV2 subtype			UniProt access.	MW [kDa]	p*I*	Score	Queries matched
								
		Fold of change		*p*-value	Fold of change		*p*-value					
**Signal transduction and neuronal activity**												
46	Calmodulin	0.1	↓	0.0024	0.2	↓	0.0005	Q96HK3	17	4.1	305	9
45	Calmodulin ^VV2^	0.3	↔	0.3233	0.3	↓	0.0014	Q96HK3	17	4.1	214	7
13	Rab GDP dissociation inhibitor α (αGDI)	0.3	↓	0.001	0.6	↓	0.0194	P31150	50	5.0	192	17
12	αGDI	0.6	↓	0.0468	0.3	↓	0.001	P31150	50	5.0	554	19
11	αGDI ^VV2^	1.2	↔	0.6475	3	^↑^	0.0155	P31150	50	5.0	766	19
16	Secernin-1 ^MM1^	0.2	↓	0.0002	0.7	↔	0.0681	Q12765	46	4.7	292	12
22	Guanine nucleotide-binding protein G(o) subunit α (GNAO1) ^MM1^	0.2	↓	0.0011	0.6	↔	0.1867	P09471	40	5.3	272	15
27	14–3-3 protein beta/alpha ^MM1^	0.2	↓	0.026	1.4	↔	0.2700	P31946	28	4.8	503	12
28	14–3-3 protein zeta/delta ^MM1^	0.4	↓	0.0126	0.2	↔	0.452	Q6P3U9	28	4.7	684	14
36	Complexin-1 ^MM1^	0.1	↓	0.017	1.0	↔	0.9808	O14810	15	4.9	172	9
25	Ketosamine-3 kinase ^VV2^	1.1	↔	0.7057	0.1	↓	0.0094	Q9HA64	34	6.8	398	13
**Cell cycle and death**												
26	Carbonyl reductase [NADPH] 1 (CBR1) ^MM1^	0.2	↓	0.0219	1.2	↔	0.6492	P16152	30	8.5	338	11
7	78 kDa glucose-regulated protein (GRP78)^VV2^	2.2	↔	0.1607	2.8	^↑^	0.0293	P11021	72	5.1	92	8
8	GRP78 ^VV2^	0.5	↔	0.0576	0.3	↓	0.0307	P11021	72	5.1	287	19
17	Nucleosome assembly protein 1-like 4 (NAP1L14)^VV2^	0.6	↔	0.2045	0.5	↓	0.0032	Q99733	43	4.6	134	7
**Cell structure and motility**												
14	Tubulin beta chain ^MM1^	0.2	↓	0.0019	0.4	↔	0.1131	P07437	50	4.8	1078	14
31	Cofilin-1 ^MM1^	0.3	↓	0.0076	2.9	↔	0.1139	Q5E9F7	18	8.2	570	8
33	Destrin ^MM1^	0.1	↓	0.0002	0.8	↔	0.3577	Q5E9D5	18	8.1	351	13
37	Profilin-2 ^MM1^	0.1	↓	0.0226	0.4	↔	0.1591	P35080	15	6.5	273	8
44	Beta-centractin ^MM1^	0.4	↓	0.0135	0.5	↔	0.078	P42025	42	5.9	282	7
32	Stathmin ^VV2^	0.7	↔	0.4257	2.6	^↑^	0.0005	Q93045	21	8.4	177	8
43	Septin-11 ^MM1^	2.5	^↑^	0.0442	1.8	↔	0.1827	Q9NVA2	49	6.4	292	14
**Protein metabolism**												
3	Heat shock protein HSP 90 (Hsp90)	0.04	↓	1.1E-06	0.1	↓	9,6E-05	P07900	85	4.9	404	23
2	Hsp90 ^MM1^	0.04	↓	0.0232	0.5	↔	0.2427	P07900	85	4.9	320	17
1	Hsp90 ↓^↑^	0.1	↓	0.0083	7.7	^↑^	0.0426	P07900	85	4.9	327	19
4	Hsp90 ↓^↑^	0.04	↓	0.0043	12.3	^↑^	0.0459	P07900	85	4.9	101	5
29	Ubiquitin carboxyl-terminal hydrolase isozyme L1 (UCHL1) ^MM1^	0.1	↓	0.0001	0.6	↔	0.081	P09936	25	5.3	440	15
34a	Small ubiquitin-related modifier 2 (SUMO 2)	0.2	↓	0.001	0.4	↓	0.043	P61956	11	5.3	77	2
34b	Small ubiquitin-related modifier 3 (SUMO 3)							P55854	11	5.3	74	2
35a	SUMO 2 ^VV2^	0.8	↔	0.0238	6.9	^↑^	0.0005	P61956	11	5.3	77	1
35b	SUMO 3 ^VV2^							P55854	11	5.3	77	1
30	Proteasome subunit beta type-4 (PSMB4) ^MM1^	4.8	^↑^	0.0002	0.5	↔	0.1523	P28070	29	5.7	186	5
9	Heat shock cognate 71 kDa protein	15.4	^↑^	0.0001	2.7	^↑^	0.0482	P11142	71	5.4	137	13
**Energy metabolism**												
10	Transketolase	0.2	↓	0.0047	0.4	↓	0.016	P29401	16	7.6	295	12
23	Alcohol dehydrogenase [NADP+] (AKR1A1) ^MM1^	0.3	↓	0.0072	0.5	↔	0.1008	P14550	36	6.3	299	12
19	4-trimethylaminobutyraldehyde dehydrogenase (ALDH9A1) ^VV2^	1.1	↔	0.4705	2.7	^↑^	0.031	P49189	54	5.7	84	4
18	ALDH9A1 ^MM1^	5.4	^↑^	0.018	2.8	↔	0.0991	P49189	54	5.7	128	8
24	Malate dehydrogenase, cytoplasmic ^VV2^	3.4	↔	0.3299	0.1	↓	0.0018	P40925	36	6.9	543	14
6	Aconitate hydratase, mitochondrial ^VV2^	2.1	↔	0.0826	0.3	↓	0.0253	Q99798	85	7.4	343	17
5	Aconitate hydratase, mitochondrial ↓^↑^	2.9	^↑^	0.0204	0.1	↓	0.0013	Q99798	85	7.4	506	17
**Other function**												
39	Hemoglobin subunit beta ^VV2^	1.5	↔	0.3874	2.5	^↑^	0.0246	Q549N7	16	6.8	464	9
38	Hemoglobin subunit beta ^MM1^	2	^↑^	0.0256	2.4	↔	0.0904	Q549N7	16	6.8	604	10
40	Hemoglobin subunit alpha ^VV2^	2.3	↔	0.0579	2.5	^↑^	0.0024	P69905	15	8.7	257	5
41	Hemoglobin subunit alpha ^VV2^	1	↔	0.9209	4.6	^↑^	0.0031	P69905	15	8.7	348	7
15a	Selenium-binding protein 1 ^VV2^	0.8	↔	0.5081	0.3	↓	0.0194	Q13228	52	5.9	133	9
15b	Cytosolic nonspecific dipeptidase ^VV2^							Q96KP4	53	5.7	113	11
20	Phytanoyl-CoA hydroxylase-interacting protein (PHYHIPL) ^VV2^	1.3	↔	0.0431	0.5	↓	0.0112	Q96FC7	42	6.0	158	7
21	NIF3-like protein ^MM1^	6.8	^↑^	0.0231	3.2	↔	0.1423	Q9GZT8	42	6.2	186	6
42	Macrophage migration inhibitory factor (MIF) ↓^↑^	2	^↑^	0.0096	0.5	↓	0.0372	P14174	12	7.7	101	3

### 3.2 Signal transduction and neuronal activity

This group includes the above mentioned calmodulin, αGDI isoforms, and several subtype-specific proteins. To the MM1-subgroup belong regulators of signal transduction, like GNAO1, and both 14–3-3 isoforms, as well as secernin-1, and complexin-1 that are involved in exocytosis. The second calmodulin isoform (ID 45), and the largely unknown ketosamine-3 kinase belong to the VV2-subgroup. All these proteins displayed lower expression.

### 3.3 Cell cycle and death

A decreased level of CBR1, which plays an important role in the detoxification of reactive lipid aldehydes, was found in the MM1 subtype. Moreover, the expression level of NAP1L1, which may be involved in chromatin formation, was reduced. One isoform of the anti-apoptotic GRP78 displayed a decreased expression level, while the second one displayed a higher level (ID 7 and ID 8, respectively). The last two mentioned proteins were differentially expressed in the VV2 subtype.

### 3.4 Cell structure and motility

In the MM1 subtype, the proteins associated with microtubule filaments (tubulin beta chain and beta-centractin), and these ones associated with actin filaments (cofilin-1, destrin, and profilin-2) showed lower expression levels. While septin-11, which is associated with cellular membranes, actin filaments, and microtubules, showed a higher one. The expression of stathmin, which is involved in the microtubule destabilization, was increased in the VV2 subtype.

### 3.5 Protein metabolism

To this group belong the abovementioned: Hsp90 isoforms, SUMO 2/3, and heat shock cognate 71 kDa protein that were differentially expressed in both subtypes. Moreover, another Hsp90 isoform (ID 2) and UCHL1, which participate in protein ubiquitination processes, were produced at the lower level, while PSMB4, a part of proteasome, was expressed at the higher level in the MM1 subtype. Another protein involved in the proteasomal ubiquitin-dependent processes, SUMO 2/3 (ID 35a/35b), was down expressed in the VV2 subtype.

### 3.6 Energy metabolism

Besides transketolase and mitochondrial aconitate hydratase, common for both subtypes, this group also includes several subtype-specific proteins. AKR1A1, abundant oxidoreductase, showed lower expression in the MM1 subtype. Additionally, both proteins involved in the tricarboxylic acid cycle (cytoplasmic malate dehydrogenase and mitochondrial aconitate hydratase isoform with ID 6) also displayed lower expression, but in the VV2 subtype. Interestingly, ALDH9A1 expressed at the higher level in both subtypes, but the isoform with ID 18 was specific for the MM1, and the isoform with ID 19 was specific for the VV2 subtype.

### 3.7 Other function

The selenium-binding protein 1/cytosolic nonspecific dipeptidase (due to the almost identical molecular weight, and pI, conclusive protein identification was not possible), PHYHIPL showed decreased expression level in the VV2, while the NIF-like protein increased one in the MM1 subtype. Moreover, the hemoglobin subunit alpha was up expressed in the VV2 and hemoglobin subunit beta in both subtypes (isoform with ID 38 in the MM1, and ones with ID 39 in VV2 subtype).

### 3.8 Verification of 2D-DIGE experiments

In order to verify the results obtained in 2D-DIGE experiments, changes in abundance of three randomly selected proteins (ALDH9A1, 14–3-3β, calmodulin, and migration inhibitory factor [MIF]) were confirmed by Western blot analysis (Supporting Information File 1).

The 2.5-fold increased expression level of ALDH9A1 was confirmed in both sCJD subtypes, when compared to the CON. The significantly lower expression of 14–3-3β was found in the MM1, while in the VV2 its level remained almost unchanged. The calmodulin immunodetection revealed the presence of different isoforms with distinct molecular weights. Variations in abundance of protein spots related to calmodulin were likely a result of the molecular weight shift in the sCJD when compared to the CON.

A not very high, but statistically significant increase in expression level of the MIF expression was detected in MM1, while a strong tendency to lower MIF production was found in VV2. Moreover, there was a 1.6-fold difference in the protein level when both subtypes were compared with each other.

All the mentioned changes concerning protein expression were significant in the one-way ANOVA test with Bonferroni correction.

### 3.9 Rab3a recycling in sCJD

Since both sCJD-associated αGDI and Hsp90 control Rab3a-mediated neurotransmitter release and Rab3a cellular localization, thus its functional state we focused on Rab3a recycling in further experiments. Dysfunction of the Rab3a recycling, thus neurotransmitter release might have an impact on the cognitive impairment observed in the sCJD.

**Figure 3:**
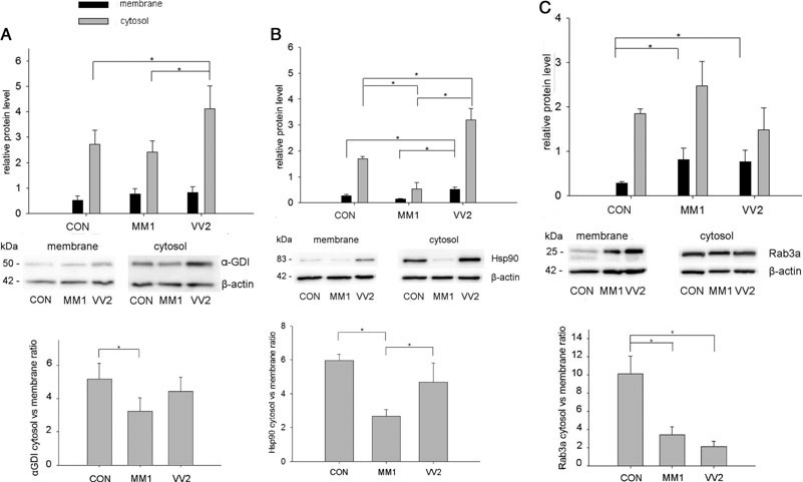
Levels and computed ratios between cytosol pool and membrane pool for αGDI (panel A), Hsp90 (panel B), and Rab3a (panel C). After separation of brain homogenates into cytosol (gray bar) and membrane fraction (black bar) level of αGDI, Hsp90, and Rab3a was semiquantified. Graphs show mean from at least three different samples and error bars represent SD. The chemiluminescence signals were normalized to the β-actin signal. The representative blots are shown below graphs. * indicates *p*-value < 0.05 in one-way ANOVA followed by Bonferroni correction.

To address the question as to whether or not Rab3a recycling is affected in the sCJD, membrane and cytosol fractions were prepared from brain homogenates. A proper and efficient fractionation was proven by Western blot analysis directed against caveolin-1, Na/K ATPase (both membrane markers), and synaptophysin (synaptic marker). Positive immunostaining was detected only in the membrane fraction (Mem), confirming that fractions of interest were properly isolated. A subsequent semiquantitative analysis of Rab3a, αGDI, and Hsp90 immunoblots was performed for both fractions, and the ratios of membrane and cytosol level were calculated as well (Fig. 3).

In the VV2 cytosol fraction, αGDI was at an approximately 1.5-fold higher level when compared to the CON and MM1 subtypes. Although αGDI differential expression in the MM1 subtype was not confirmed by Western blot, its cytosol versus membrane ratio was significantly lower. In the membrane fractions of both analyzed subtypes, the αGDI level was slightly, but not significantly increased (Fig. 3A).

In the MM1 subtype, the Hsp90 level was 3.2-fold and 1.9-fold lower in cytosol and the membrane fraction, respectively. In turn, in the VV2 subtype, the Hsp90 level was 1.8-fold higher in both fractions. Similarly to αGDI, the Hsp90 ratio of the cytosol pool versus the membrane pool was (2-fold) lower only in the MM1 subtype (Fig. 3B).

Interestingly, an approximately 2.8-fold accumulation of the Rab3a in the membrane fraction and a decreased cytosol versus membrane ratio were found in both sCJD subtypes analyzed (Fig. 3C).

The αGDI, Hsp90, and Rab3a demonstrated distinct patterns of differential expression in the MM1 and VV2 subtypes. In the MM1 subtype, all three proteins showed a significantly lower cytosol versus membrane ratio compared to the other sCJD subtype and the control group, as well. In the VV2 subtype, elevated levels of αGDI and Hsp90 were accompanied by an unchanged cytosol versus membrane ratio. While the Rab3a cytosol versus membrane ratio was 4.3-fold lower in the VV2 subtype compared to the control group.

## 4 Discussion

### 4.1 Subtype-specific proteome alterations in sCJD

The sCJD has an unusual degree of phenotypic heterogeneity. Based on observations that a certain disease phenotype is associated with prion type and codon 129 genotype, six different sCJD subtypes (MM1, MM2, MV1, MV2, VV1, and VV2) were distinguished. In this study, the two most frequent ones (MM1 and VV2) were analyzed for proteome changes using a differential proteomic approach.

The MM1 subtype is a myoclonic type with cognitive impairment accompanied by mental and visual signs at the onset of disease. In turn, the VV2 subtype is an ataxic type, where dementia usually develops later in the progression of the disease. Differences between subtypes are also clearly found in the histopathology of the brain. For instance, the topography of the MM1-associated lesions shows that the rostral are more severely affected than the caudal brain regions, while in the VV2, it is other way around 2. Such a high degree of heterogeneity might suggest the involvement of different molecular pathways in the sCJD pathogenesis, depending on which infectious prion strain is involved 10. The proteomic data we obtained support this hypothesis.

Out of the 34 differentially expressed proteins identified, only seven showed a common alteration in both analyzed sCJD subtypes. The remaining proteins displayed a subtype-specific difference in the expression level. Interestingly, some of them, such as aconitate hydratase or macrophage migration inhibitory factor, showed a lower expression level in the MM1, while they showed a higher one in the VV2 subtype.

Since both codon 129 genotype and PrP^Sc^ type have an influence on the expression level of the brain protein, one could ask if codon 129 genotype alone may also influence the state of the brain proteome. We screened expression levels of sCJD differentially expressed proteins in the control group, which consists of three different codon 129 genotypes (MM, MV, and VV), and we did not find significant differences between genotypes for almost all of the proteins. Three proteins are the exception: septin-11, and both 14–3-3 isoforms. It seems that the presence of methionine in the codon 129 is associated with an increased level of septin-11, and the presence of valine in the codon 129 is associated with an increased level of 14–3-3. This fact might explain why these proteins did not fulfill the significance requirement in the statistical evaluation.

The sCJD-related proteins identified in this study can be classified in a few different functional blocks. The most striking difference between subtypes was found within proteins involved in the cell cycle and death, as well as in ones related to the cell structure and motility.

Supporting Information File 2 provides a detailed description of the biological function of sCJD-related proteins, as well some already known links to prion pathophysiology or other neurodegenerative processes.

Two independent studies showed that four major human prion strains largely correlate with widely accepted sCJD classification into six subtypes: MM1 + MV1, MV2 + VV2, MM1, and VV2 10,11. It is likely that these prion strains are characterized by different conformation of PrP^Sc^, which may affect interactions of cellular prion protein with PrP^Sc^ and/or other molecular partners in a way that different molecular pathways are involved in the pathogenesis of prion diseases. For instance, MM1-sCJD-associated cofilin-1, which regulates actin cytoskeleton dynamics, is a known interactor of both cellular prion protein and PrP^Sc^ 12,13. The disrupted interaction between cofilin-1 and PrP might cause the formation of actin rods, which leads to cellular transport deficits and synaptic dysfunction, as it is observed in other neurodegenerative diseases (reviewed in 14).

### 4.2 Rab3a recycling in sCJD

Synaptic vesicle exocytosis serves as the nervous system's main form of cell-to-cell communication, and it is a multi-step, tightly regulated mechanism. Modifications to any of these steps may change the strength of synaptic connections or their integrity. Growing evidence indicates that synaptic dysfunction is a key process in the development of many neurodegenerative disorders, including sCJD. Furthermore, it is very likely that synaptic failure and loss already occur before the onset of the disease. For instance, scrapie-infected mice show changes in motivational behavior long before the appearance of motor signs, and this correlates with the initial loss of presynaptic terminals in the dorsal hippocampus 15,16.

Our proteomic data indicated that some of the sCJD-related proteins are involved in signal transduction and neuronal activity. Interestingly, for almost all of them, a decreased level of expression was found in both investigated subtypes. The exception is αGDI in the VV2 subtype, where one out of three differentially expressed isoforms detected on 2D maps showed 3-fold upregulation. Due to some posttranslational modification, most probably phosphorylation 17,18, these isoforms vary in their isoelectric point, while molecular weight remains the same. Therefore, 1D analysis via Western blot revealed only one band confirming a slight but significant increase in the αGDI level when compared to the control group.

αGDI regulates the function of several Rab proteins, including Rab3a 19,20, which is crucial for the Ca^2+^-dependent exocytosis of synaptic vesicles 21,22. Rabs are functionally active when located on the membrane, but they are inactive when associated with GDI in the cytoplasm. Thus, Rab distribution between membranes and cytoplasm indicates its functional state. Therefore, we investigated Rab3a levels in both the membrane and the cytosol fraction, and we found that the pool of membrane-bound Rab3a is significantly increased in both sCJD subtypes. This suggests that either Rab3a is accumulating on the membrane due to its altered cycling or that there is some compensation mechanism that increases levels of the active Rab3a form. Since αGDI was also identified as a sCJD-related protein, we tended to assume that disrupted αGDI-mediated Rab3a recycling may cause Rab3a accumulation on the membrane.

It has been already shown that the major pool of the cytosolic αGDI form is mainly in a complex with Rab3a, while the minor pool of membrane-associated αGDI forms a complex with chaperons, including Hsp90. Moreover, αGDI interaction with the Hsp90-containing chaperone complex is required for efficient Rab3a recovery from the membrane and Ca^2+^-dependent neurotransmitter release 23,24. In our studies, we observed subtype-specific differential expression of the Hsp90. In both, cytosol and membrane of the VV2 subtype, a highly elevated Hsp90 level positively correlates with an elevated αGDI level. In the case of the MM1 subtype, a decreased level was found in both fractions. Moreover, αGDI and Hsp90 cytosol versus membrane ratios were significantly lower in the MM1 when compared to the control group and the other sCJD subtypes. This suggests an altered cellular distribution, which might also affect their biological activity.

It should be mentioned that these subtype-specific alterations of Rab3a recycling could be influenced by other factors, which were not analyzed in these studies. Moreover, using our approach we were not able to show whether only a particular isoform of αGDI or Hsp90, or rather all of them are involved in Rab3a recycling.

## 5 Concluding remarks

In the present study work, we showed alterations of the human frontal cortex proteome in two different sCJD subtypes. Striking differences in altered proteome patterns between subtypes might support the hypothesis that depending on the PrP^Sc^ form, different molecular partners/pathways could be involved in the pathogenesis of sCJD. This possibly underlies the observed clinical and pathological heterogeneity of the disease.

Furthermore, we demonstrated that Rab3a recycling accumulates on the membrane in sCJD. The dysfunction of Rab3a-mediated synaptic vesicles might contribute to the cognitive impairment of patients suffering from sCJD. The precise mechanisms underlying synaptic dysfunction in sCJD should be an issue for future studies.

## 7 Supporting information

Three Supporting Information files are provided. Supporting Information File 1 contains confirmation of proper separation between cytosol and membrane as well verification of 2D-DIGE experiments. Supporting Information File 2 gives a detailed description of the biological function of sCJD-related proteins as well some already known link to prion pathophysiology or other neurodegenerative processes. Supporting Information File 3 contains MS data including MS/MS spectra for proteins identified from three or less peptides.

## Abbreviations

αGDI: Rab GDP dissociation inhibitor alpha
AKR1A1: alcohol dehydrogenase [NADP+]
ALDH9A1: 4-trimethylaminobutyraldehyde dehydrogenase
CBR1: carbonyl reductase [NADPH1]
CON: control
GNAO1: guanine nucleotide-binding protein G(o) subunit alpha
GRP78: 78 kDa glucose-regulated protein
MIF: macrophage migration inhibitory factor
MM1: MM2, MV1, MV2, VV1, and VV2 - sCJD subtypes
PHYHIPL: phytanoyl-CoA hydrolase-interacting protein
*PRNP*: prion protein gene
PrP^Sc^: scrapie prion protein
PSMB4: proteasome subunit beta type-4
sCJD: sporadic Creutzfeldt–Jakob disease
SUMO: small ubiquitin-related modifier
UCHL1: ubiquitin carboxyl-terminal hydrolase isozyme L1

## Supplementary material

Disclaimer: Supplementary materials have been peer-reviewed but not copyedited.
