# Supplementary material for: Sporadic Creutzfeldt–Jakob disease subtype-specific alterations of the brain proteome: Impact on Rab3a recycling
Source: Proteomics. 2012 Dec 12;12(23-24):3610–20. doi: 10.1002/pmic.201200201 (PMC3565451; doi:10.1002/pmic.201200201)

**Efficiency of separation between membrane (Mem) and cytosol (Cyt) faction shown by Western blot analysis directed against caveolin 1 and Na/K ATPase (membrane-specific proteins) and synaptophysin (synaptic marker)**

**
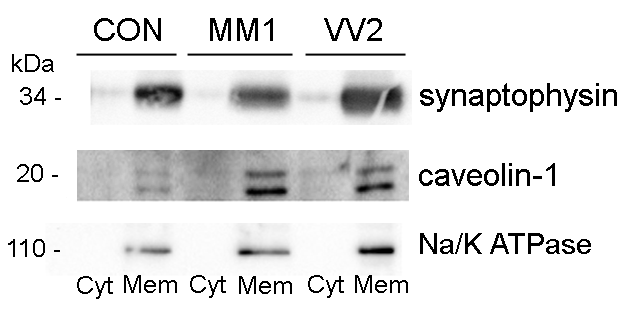
**

**Verification of 2D-DIGE experiments**

The 2.5-fold up-regulation of ALDH9A1 expression was confirmed in both sCJD subtypes, when compared to age-matched control group (CON) (Panel A).

The significant down-regulation of 14-3-3β expression was found in MM1, while in VV2 its level remained almost unchanged (Panel B).

The camodulin was detected by mutli-Western blot bands. The differences in abundance of protein spots related to camodulin are most probably result of the shift of molecular weight in sCJD in comparison to CON (Panel C).

Not very high, but statistically significant up-regulation of the macrophage migration inhibitory factor (MIF) expression was detected in MM1, while strong tendency to lower MIF production was found in VV2. Moreover, there was 1.6-fold difference in the protein level when both subtypes were compared with each other (Panel D).

All mentioned above changes in protein expression were significant in one way ANOVA test.


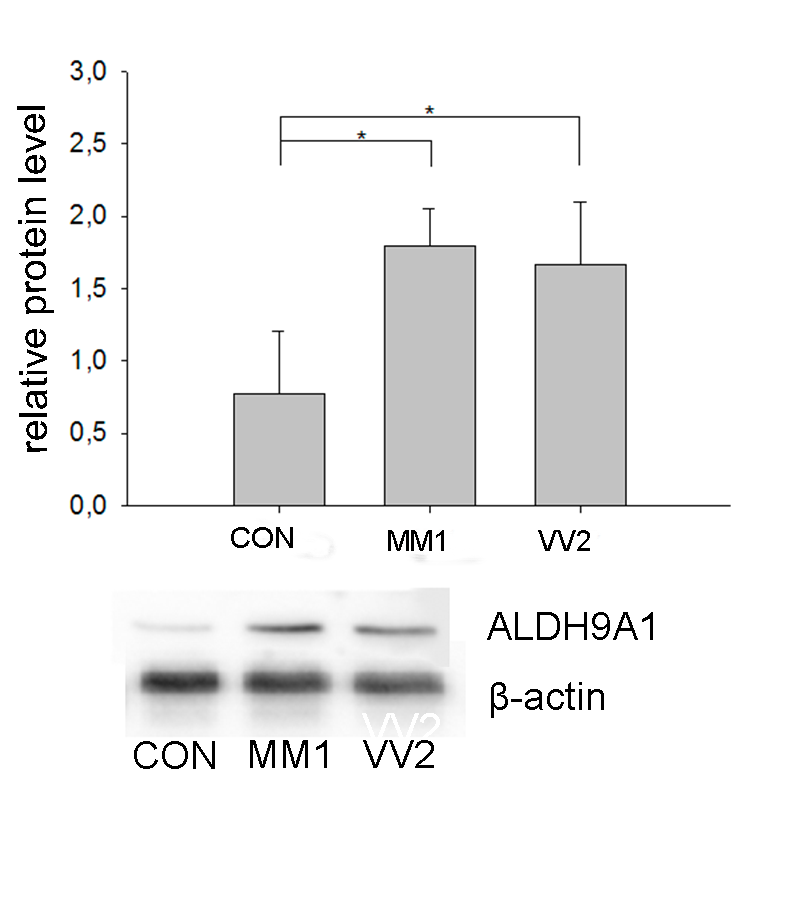
A


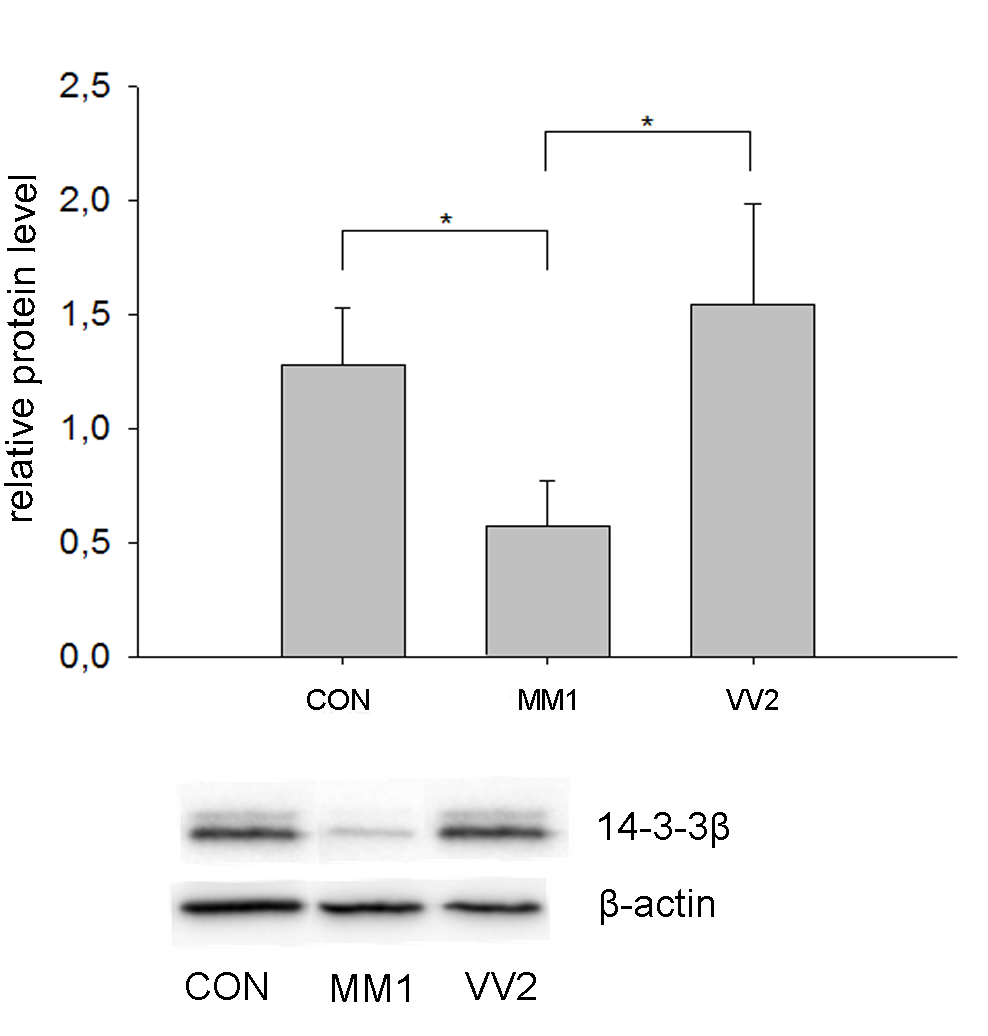
B


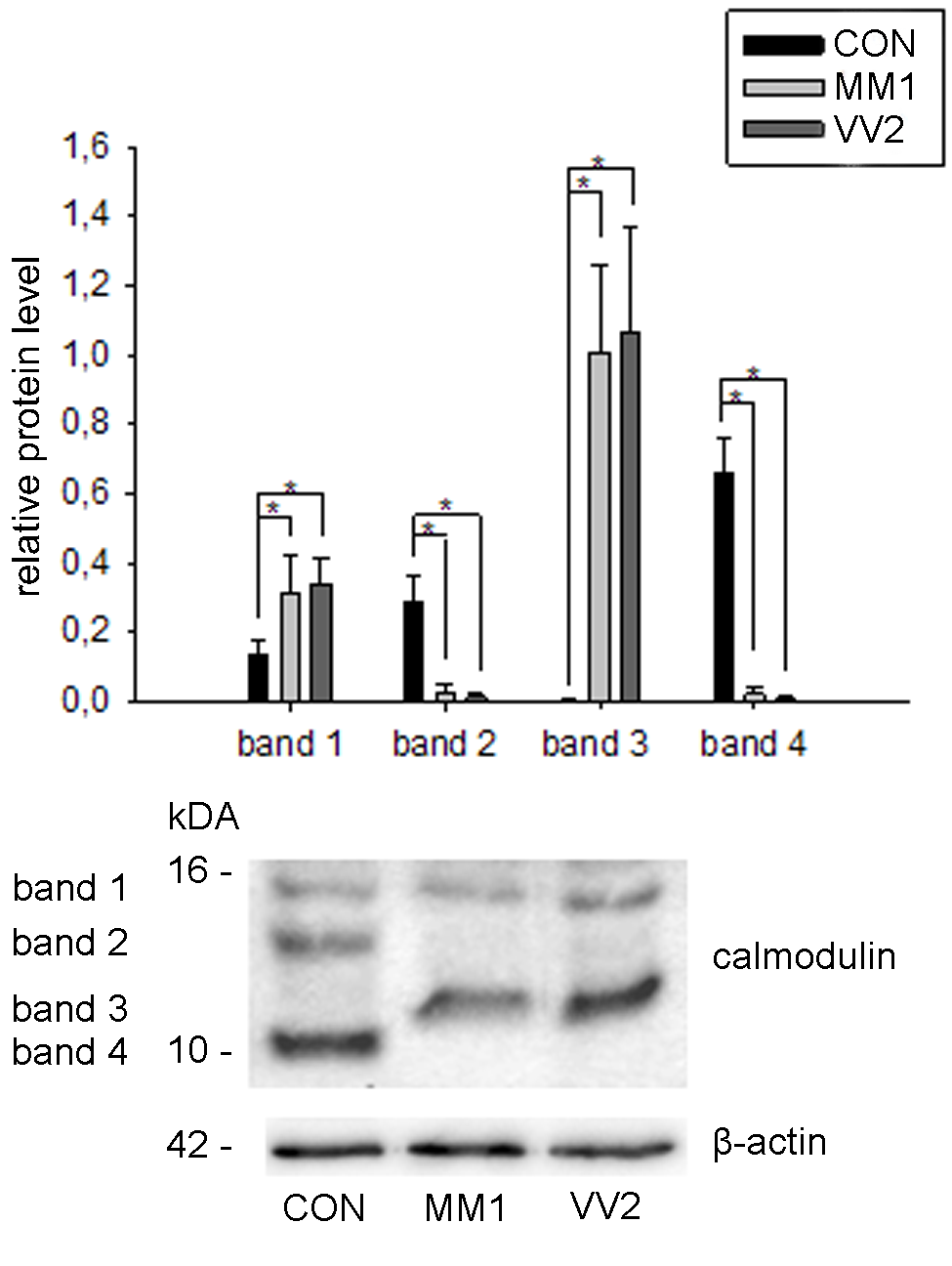


C

D


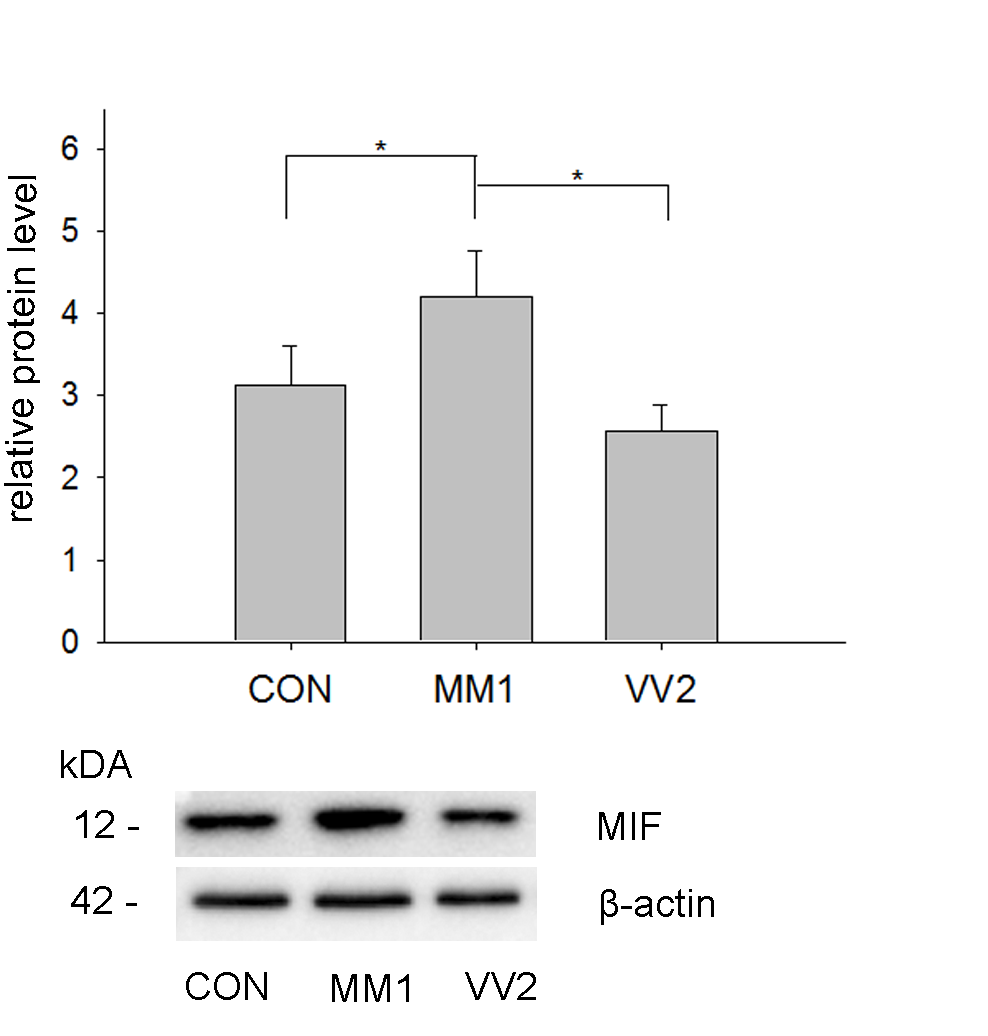

Supplement: Supplementary file 1 [file pmic0012-3610-SD1.doc]
