# Supplementary material for: Sporadic Creutzfeldt–Jakob disease subtype-specific alterations of the brain proteome: Impact on Rab3a recycling
Source: Proteomics. 2012 Dec 12;12(23-24):3610–20. doi: 10.1002/pmic.201200201 (PMC3565451; doi:10.1002/pmic.201200201)
Supplement: Supplementary file 2 [file pmic0012-3610-SD2.doc]

| ID | Protein name | Function and known links to prion pathobiology  or other neurodegenerative processes |
| --- | --- | --- |
| 1-4 | Heat shock protein  HSP 90 (Hsp90) | Molecular chaperone involved in stress response and apoptosis. Hsp90 was identified as interacting partner of human PrP^C^ using One-STrEP-tag technology [1] and FLAG-tagged PrP in neuroblastoma cell-based disease model studies of PrP molecular network [2], co-purifies with the protease-resistant core of PrP (PrP27-30) extracted from the 263K-infected hamster brains [3]. Down-regulation of Hsp90 expression was found in hippocampus of patients affected with schizophrenia and bipolar disorders [4]. |
| 5-6 | Aconitate hydratase, mitochondrial | Aconitate hydratase is involved in tricarboxylic acid cycle. Its production was down-regulated when PrP with D178N/Met129 mutation, which is associated with fatal familial insomnia (FFI) was expressed in N2a cells [5]. |
| 7-8 | 78 kDa glucose-regulated protein (Grp78, BiP) | BiP is involved in glucose starvation response, anti-apoptosis and cell survival. BiP is an interaction partner of PrP in the living brain [6], fully functional myc-tagged PrP in supramolecular complex studies [7] and human PrP^C^ using One-STrEP-tag technology [1]. Moreover, BiP can bind to mutant PrP (Q217R mutation associated with GSS) and mediates its degradation by proteasome [8]. |
| 9 | Heat shock cognate  71 kDa protein (Hsc70) | Molecular chaperone involved in stress response and apoptosis. Hsc70 binds PrP in pH- und copper-dependent manner [9]. Hsc70 overexpression was found in diencephalons of sheep naturally infected with scrapie [10]. |
| 10 | Transketolase | Transketolase is related to pentose phosphate pathway. Its elevated level was found in CSF of patients with sCJD [11]. Its production was down-regulated when PrP with D178N/Met129 mutation, which is associated with fatal familial insomnia (FFI), was expressed in N2a cells [5]. |
| 11-13 | Rab GDP dissociation inhibitor α (αGDI) | αGDI regulates the GDP/GTP exchange reaction of most Rab proteins, thus involved in synaptic signal transduction. The expression of PrP with FFI-associated D178N mutation in neuroblastoma N2a cells caused alteration of αGDI/Rab11 pathway [5]. |
| 14 | Tubulin beta chain | Tubulin is a major constituent of microtubules. It is an interaction partner of fully functional myc-tagged PrP in supramolecular complex studies [6] and PrP^C^ [12], and co-purifies with the protease-resistant core of PrP (PrP27-30) extracted from the 263K-infected hamster brains [3]. Its expression level was decreased in global studies of cerebral gene expression profile upon CJD-infection [13]. Moreover, down-regulation of tubulin expression was also found in hippocampus of patients affected with schizophrenia [4]. |
| 15a | Selenium-binding  protein 1 (SBP1) | Selenium-binding protein 1 might be involved in intra-Golgi protein transport. SBP1 is elevated in the brain and blood of patients with schizophrenia [14]. |
| 15b | Cytosolic non-specific dipeptidase | Cytosolic non-specific dipeptidase is involved in proteolysis and preferentially hydrolyses hydrophobic dipeptides. |
| 16 | Secernin-1 | Secernin-1 regulates exocytosis. It is an interaction partner of fully functional myc-tagged PrP in supramolecular complex studies [6]. |
| 17 | Nucleosome assembly protein 1-like 4 (NAP1L1) | NAP1L1 might be involved in modulation of chromatin formation and might contribute to regulation of cell proliferation. |
| 18-19 | 4-trimethylaminobutyr-  aldehyde dehydrogenase (ALDH9A1) | ALDH9A1 is involved in neurotransmitter, hormone, aldehyde and carnitine biosynthetic process. |
| 20 | Phytanoyl-CoA hydroxylase-interacting protein ( **PHYHIPL)** | **PHYHIPL** might play a role in the development of the central system. Its expression was altered upon global ischemic condition in *APOE* transgenic mice [15]. |
| 21 | NIF3-like protein | NIF3-like protein is a transcription binding factor. |
| 22 | Guanine nucleotide-binding protein G(o) subunit alpha (GNAO1) | GNAO1 is modulator or transducer in various transmembrane signaling systems. It is an interaction partner of FLAG-tagged PrP in cell-based disease model studies of PrP molecular network [2]. GNAO1 expression level was decreased in global studies of cerebral gene expression profile upon CJD-infection [13]. |
| 23 | Alcohol dehydrogenase [NADP+]  ( Aldehyde reductase, **AKR1A1**) | **AKR1A1 is involved in** glucose metabolic process, aldehyde catabolic process and oxidation reduction. |
| 24 | Malate dehydrogenase, cytoplasmic | Malate dehydrogenase is involved in tricarboxylic acid cycle. It was identified as an interacting partner of human PrP^C^ using One-STrEP-tag technology [1]. Its elevated level was found in CSF of patients with sCJD [10]. |
| 25 | Ketosamine-3 kinase | Ketosamine-3 kinase might be responsible for the removal of fructosamines from proteins. |
| 26 | Carbonyl reductase [NADPH] 1 (CBR1) | CBR1 plays an important role in the detoxification of reactive lipid aldehydes. Mutation in the gene encoding a homologue of CBR1 causes oxidative stress-induced neurodegeneration in *Drosophila melanogaster* [16]. Moreover, CBR1 overexpression of the human CBR1 in NIH3T3 cells protects from ROS-induced cellular damage [17]. |
| 27 | 14-3-3 protein beta/alpha | 14-3-3 proteins are adapter proteins implicated in the regulation of a large spectrum of signaling pathways. They bind to a large number of partners, usually by recognition of a phosphoserine or phosphothreonine motif. They regulate neuronal differentiation, signal transduction and synaptic plasticity. PrP^C^ was coimmunoprecipitated with 14-3-3 proteins in the human brain protein extract [18]. 14-3-3 protein beta/alpha expression level was decreased in global studies of cerebral gene expression profile in sCJD [13]. 14-3-3 protein zeta/delta was identified as interacting partner of human PrP^C^ using One-STrEP-tag technology [1]. 14-3-3 epsilon and zeta/delta were identified as differentially regulated in murine model of inherited prion disease associated with nine-octapeptide insertion in PrP [19]. Moreover, 14-3-3 proteins are CSF biomarker for differential diagnosis of CJD [20,21]. Down-regulation of 14-3-3 proteins expression was also found in hippocampus of patients affected with schizophrenia [4]. |
| 28 | 14-3-3 protein zeta/delta |
| 29 | Ubiquitin carboxyl-terminal hydrolase isozyme L1 (UCHL1) | UCHL1 is involved in protein ubiquitination processes and cell death. Oxidative modifications and down-regulation of UCHL1 is associated with idiopathic Parkinson's and Alzheimer's diseases (AD) [22]. Administration of a UCHL1 fusion protein to supplement endogenous UCHL1 had a protective effect on memory loss in AD mouse model [23]. Its expression is down-regulated in frontotemporal lobar degeneration with ubiquitinated inclusions [24]. Moreover, proteomic studies showed decreased level of UCHL1 in entorhinal cortex in AD [25]. Down-regulation of UCHL1 expression was also found in hippocampus of patients affected with schizophrenia [4]. |
| 30 | Proteasome subunit  beta type-4 (PSMB4) | PSMB4 is part of proteasome, which regulates cycle cycle and apoptotic processes. |
| 31 | Cofilin-1 | Cofilin-1 controls actin polymerization and depolymerisation and is a major component of intranuclear and cytoplasmic actin rods. It is an interacting partner of human PrP^C^ using One-STrEP-tag technology and fully functional myc-tagged PrP in supramolecular complex studies [6], and co-purifies with the protease-resistant core of PrP (PrP27-30) extracted from the 263K-infected hamster brains [3]. Destrin/cofilin inclusions were found in human AD brain and overlie amyloid plaques. Mediators of neurodegeneration (ischemia, oxidative stress and excitotoxicity) induce rapid formation of transient or persistent rod-like inclusions containing destrin/cofilin and actin in axons and dendrites of cultured hippocampal neurons [26]. |
| 32 | Stathmin | Stathmin, a tubulin-binding protein regulated by phosphorylation at four serine residues, is involved in regulation of microtubule filament system by destabilization of microtubules. PrP^C^ clustering at the surface of GT1-7 cells caused stathmin phosphorylation at serine 16, what is essential for regulation of stathmin microtubule-destabilizing activity [27, 28]. Up-regulation of stathmin expression was found in hippocampus of patients affected with schizophrenia and bipolar disorder [4]. |
| 33 | Destrin | Destrin is actin-depolymerizing protein and severs actin filaments. Destrin/cofilin inclusions were found in human AD brain and overlie amyloid plaques. Mediators of neurodegeneration (ischemia, oxidative stress and excitotoxicity) induce the rapid formation of transient or persistent rod-like inclusions containing destrin/cofilin and actin in axons and dendrites of cultured hippocampal neurons [26]. |
| 34-35a | Small ubiquitin-related modifier 2/3  (SUMO 2 and SUMO3) | SUMO2 and 3 regulate proteasomal ubiquitin-dependent protein catabolic process. SUMO2 and 3 are activated in cultured cells exposed to various stress conditions, including anoxic conditions, hypothermia, and hypoxia [29]. Activation of SUMO2/3 conjugation is an endogenous neuroprotective stress response in primary cortical neurons [30]. |
| 34-35b |
| 36 | Complexin-1 | Complexin-1 positively regulates a late step in synaptic vesicle exocytosis and prevents SNARE complex from releasing neurotransmitters until an action potential arrives at the synapse. Its protein and gene expression level are decreased in AD brain [31, 32]. In the rat model, traumatic brain injury was associated with initial increase and subsequent decrease of complexin-1 level [33]. |
| 37 | Profilin-2 | Profilin-2 is involved in actin cytoskeleton organization. Its expression level found to be decreased in global studies of cerebral gene expression profile in sCJD [13]. However, expression of profilin 2 was significantly increased in brains from 12 month-old aged senescence-accelerated mice following administration of antisense oligonucleotide directed at the Aβ region of amyloid precursor protein [34]. Moreover, proteomic studies showed decreased level of profilin 2 in cerebellum in AD [25]. |
| 38-39 | Hemoglobin subunit beta (HBB) | Hemoglobin is involved in oxygen and nitric oxide transport, hydrogen peroxide catabolic process and regulation of cell death. Reduced level of HBA and HBB was found in neurons with punctuate hyperphosphorylated tau deposits and tangles in the hippocampus and frontal cortex in AD; in ballooned neurons containing αβ-crystallin in the amygdala in AD and argyrophilic grain disease; and in neurons with punctuate α-synuclein deposits and in neurons with Lewy bodies in the substantia nigra pars compacta and in vulnerable neurons of the medulla oblongata in PD and DLB [35]. Proteomic studies showed increased and decreased level of HBB, depending on brain region in AD [25]. Moreover, hemoglobin promotes Aβ oligomer formation and localizes in neurons and amyloid deposits [36]. |
| 40-41 | Hemoglobin subunit alpha (HBA) |
| 42 | Macrophage migration inhibitory factor (MIF) | MIF, a pro-inflammatory cytokine, participates in regulation od DNA damage response, apoptotic processes and cell aging. Proteomic studies showed decreased level of MIF entorhinal cortex region in AD [25]. MIF elevated level was found in CSF and it was proposed as AD biomarker [37,38,39]. Moreover, MIF is associated with Aβ deposits in APP 23 mice, thus it might be involved in plaque formation and in the inflammatory process surrounding the plaques [40]. |
| 43 | Septin-11 | Septin-11 is filament-forming cytoskeletal GTPase, which might play a role in the cytoarchitecture of neurons. It is also involved in cell cycle and junction. Tau oligomers injected into mice caused synaptic dysfunction and reduction of septin-11 level [41]. The insoluble septin-11 accumulates in cortex of patients affected with the frontotemporal lobar degeneration with ubiquitin-immunoreactive inclusions [42]. Moreover, down-regulation of septin-11 expression was also found in hippocampus of patients affected with schizophrenia and bipolar disorder [4]. |
| 44 | Beta-centractin | Component of a multi-subunit complex involved in microtubule based vesicle motility and is associated with centrosome. |
| 45-46 | Calmodulin | Through Ca^2+^, calmodulin mediates the control of a large number of enzymes (including kinases and phosphatases), ion channels and other proteins. Elevated level of calmodulin was found in cerebellum in AD [43]. Moreover, it regulates non-amyliodogenic processing of amyloid precursor protein [44]. Calmodulin and Ca^2+^-binding proteins are implicated in AD pathogensis (reviewed in [45]). |

**Bibliography**

1. Zafar, S., von Ahsen, N., Oellerich, M., Zerr, I., Schulz-Schaeffer, W.J., Armstrong, V.W., Asif, A.R., Proteomics approach to identify the interacting partners of cellular prion protein and characterization of Rab7a interaction in neuronal cells. *J Proteome Res* 2011, *10*, 3123-3135.
2. Watts, J.C., Huo, H., Bai, Y., Ehsani, S., Jeon, A.H., Shi, T., Daude, N., Lau, A., Young, R., Xu, L., Carlson, G.A., Williams, D., Westaway, D., Schmitt-Ulms, G., Interactome analyses identify ties of PrP and its mammalian paralogs to oligomannosidic N-glycans and endoplasmic reticulum-derived chaperones. *PLoS Pathog* 2009, *5*, e100060.
3. Giorgi, A., Di Francesco, L., Principe, S., Mignogna, G., Sennels, L., Mancone, C., Alonzi, T., Sbriccoli, M., De Pascalis, A., Rappsilber, J., Cardone, F., Pocchiari, M., Maras, B., Schininà, M.E., Proteomic profiling of PrP27-30-enriched preparations extracted from the brain of hamsters with experimental scrapie. *Proteomics* 2009, *9*, 3802-3814.
4. Föcking, M., Dicker, P., English, J.A., Schubert, K.O., Dunn, M.J., Cotter, D.R., Common proteomic changes in the hippocampus in schizophrenia and bipolar disorder and particular evidence for involvement of cornu ammonis regions 2 and 3. *Arch Gen Psychiatry* 2011, *68*, 477-488.
5. Massignan, T., Biasini, E., Lauranzano, E., Veglianese, P., Pignataro, M., Fioriti, L., Harris, D.A., Salmona, M., Chiesa, R., Bonetto, V., Mutant prion protein expression is associated with an alteration of the Rab GDP dissociation inhibitor alpha (GDI)/Rab11 pathway. *Mol Cell Proteomics* 2010, *9*, 611-622.
6. Schmitt-Ulms, G., Hansen, K., Liu, J., Cowdrey, C., Yang, J., DeArmond, S.J., Cohen, F.E., Prusiner, S.B., Baldwin, M.A., Time-controlled transcardiac perfusion cross-linking for the study of protein interactions in complex tissues. *Nat Biotechnol* 2004, *22*, 724-731.
7. Rutishauser, D., Mertz, K.D., Moos, R., Brunner, E., Rülicke, T., Calella, A.M., Aguzzi, A., The comprehensive native interactome of a fully functional tagged prion protein. *PLoS One* 2009, *4*, e4446.
8. Jin, T., Gu, Y., Zanusso, G., Sy, M., Kumar, A., Cohen, M., Gambetti, P., Singh, N., The chaperone protein BiP binds to a mutant prion protein and mediates its degradation by the proteasome. *J Biol Chem* 2000, *275*, 38699-38704.
9. Wilkins, S., Choglay, A.A., Chapple, J.P., van der Spuy, J., Rhie, A., Birkett, C.R., Cheetham, M.E., The binding of the molecular chaperone Hsc70 to the prion protein PrP is modulated by pH and copper. *Int J Biochem Cell Biol* 2010, *42*, 1226-1232.
10. Serrano C., Bolea, R., Lyahyai, J., Filali, H., Varona, L., Marcos-Carcavilla, A., Acín, C., Calvo, J.H., Serrano, M., Badiola, J.J., Zaragoza, P., Martín-Burriel, I., Changes in HSP gene and protein expression in natural scrapie with brain damage. *Vet Res* 2011, *42*, 13.
11. Gawinecka, J., Dieks, J., Asif, A.R., Carimalo, J., Heinemann, U., Streich, J.H., Dihazi, H., Schulz-Schaeffer, W., Zerr, I., Codon 129 polymorphism specific cerebrospinal fluid proteome pattern in sporadic Creutzfeldt-Jakob disease and the implication of glycolytic enzymes in prion-induced pathology. *J Proteome Res* 2010, *9*, 5646-5657.
12. Nieznanski, K., Nieznanska, H., Skowronek, K.J., Osiecka, K.M., Stepkowski, D., Direct interaction between prion protein and tubulin. *Biochem Biophys Res Commun* 2005, *334*, 403-411.
13. Xiang, W., Windl, O., Westner, I.M., Neumann, M., Zerr, I., Lederer, R.M., Kretzschmar, H.A., Cerebral gene expression profiles in sporadic Creutzfeldt-Jakob disease. *Ann Neurol* 2005, *58*, 242-257.
14. Glatt, S.J., Everall, I.P., Kremen, W.S., Corbeil, J., Sásik, R., Khanlou, N., Han, M., Liew, C.C., Tsuang, M.T., Comparative gene expression analysis of blood and brain provides concurrent validation of SELENBP1 up-regulation in schizophrenia. *Proc Natl Acad Sci U S A* 2005, *102*, 15533-15538.
15. James, R., Searcy, J.L., Le Bihan, T., Martin, S.F., Gliddon, C.M., Povey, J., Deighton, R.F., Kerr, L.E., McCulloch, J., Horsburgh, K., Proteomic analysis of mitochondria in APOE transgenic mice and in response to an ischemic challenge. *J Cereb Blood Flow Metab* 2012, *32*, 164-176.
16. Botella, J.A., Ulschmid, J.K., Gruenewald, C., Moehle, C., Kretzschmar, D., Becker, K., Schneuwly, S., The Drosophila carbonyl reductase sniffer prevents oxidative stress-induced neurodegeneration*. Curr Biol* 2004, *14*, 782-786.
17. Kelner, M.J., Estes, L., Rutherford, M., Uglik, S.F., Peitzke, J.A. Heterologous expression of carbonyl reductase: demonstration of prostaglandin 9-ketoreductase activity and paraquat resistance. *Life Sci* 1997, *61*, 2317-2322.
18. Satoh, J., Onoue, H., Arima, K., Yamamura, T., The 14-3-3 protein forms a molecular complex with heat shock protein Hsp60 and cellular prion protein. *J Neuropathol Exp Neurol* 2005, *64*, 858-886.
19. Biasini, E., Massignan, T., Fioriti, L., Rossi, V., Dossena, S., Salmona, M., Forloni, G., Bonetto, V., Chiesa, R., Analysis of the cerebellar proteome in a transgenic mouse model of inherited prion disease reveals preclinical alteration of calcineurin activity. *Proteomics* 2006, *6*, 2823-2834.
20. Hsich, G., Kenney, K., Gibbs Jr., C.J., Lee, K.H., Harrington, M.G., The 14-3-3 brain protein in cerebrospinal fluid as a marker for transmissible spongifrom encephalopathies. *N Engl J Med* 1996, *335*, 924-930.
21. Zerr, I., Bodemer, M., Otto, M., Poser, S., Windl, O., Kretzschmar, H.A., Gefeller, O., Weber, T., Diagnosis of Creutzfeldt-Jakob disease by two-dimensional gel electrophoresis of cerebrospinal fluid. *Lancet* 1996, *348*, 846-849.
22. Choi, J., Levey, A.I., Weintraub, S.T., Rees, H.D., Gearing, M., Chin, L.S., Li, L., Oxidative modifications and down-regulation of ubiquitin carboxyl-terminal hydrolase L1 associated with idiopathic Parkinson's and Alzheimer's diseases. *J Biol Chem* 2004, *279*, 13256-13264.
23. Gong. B., Cao, Z., Zheng, P., Vitolo, O.V., Liu, S., Staniszewski, A., Moolman, D., Zhang, H., Shelanski, M., Arancio, O., Ubiquitin hydrolase Uch-L1 rescues beta-amyloid-induced decreases in synaptic function and contextual memory. *Cell* 2006, *12*, 775-88.
24. Mishra, M., Paunesku, T., Woloschak, G.E., Siddique, T., Zhu, L.J., Lin, S., Greco, K., Bigio, E.H., Gene expression analysis of frontotemporal lobar degeneration of the motor neuron disease type with ubiquitinated inclusions. *Acta Neuropathol* 2007, *114*, 81-94.
25. Schonberger, S.J., Edgar, P.F., Kydd, R., Faull, R.L., Cooper, G.J., Proteomic analysis of the brain in Alzheimer's disease: molecular phenotype of a complex disease process. *Proteomics* 2001, *1*,1519-1528.
26. Minamide, L.S., Striegl, A.M., Boyle, J.A., Meberg, P.J., Bamburg, J.R., Neurodegenerative stimuli induce persistent ADF/cofilin-actin rods that disrupt distal neurite function. *Nat Cell Biol* 2000, *2*, 629-636.
27. Melander Gradin, H., Marklund, U., Larsson, N., Chatila, T.A., Gullberg, M., Regulation of microtubule dynamics by Ca2+/calmodulin-dependent kinase IV/Gr-dependent phosphorylation of oncoprotein 18. *Mol Cell Biol* 1997, *17*, 3459-3467.
28. Monnet, C., Gavard, J., Mège, R.M., Sobel, A., Clustering of cellular prion protein induces ERK1/2 and stathmin phosphorylation in GT1-7 neuronal cells. *FEBS Lett* 2004, *576*, 114-118.
29. Lee, Y.J., Castri, P., Bembry, J., Maric, D., Auh, S., Hallenbeck, J.M., SUMOylation participates in induction of ischemic tolerance. *J Neurochem* 2009, *109*, 257-267.
30. Datwyler, A.L., Lättig-Tünnemann, G., Yang, W., Paschen, W., Lee, S.L., Dirnagl, U., Endres, M., Harms, C., SUMO2/3 conjugation is an endogenous neuroprotective mechanism. *J Cereb Blood Flow Metab* 2011, *31*, 2152-2159.
31. Tannenberg, R.K., Scott, H.L., Tannenberg, A.E., Dodd, P.R., Selective loss of synaptic proteins in Alzheimer's disease: evidence for an increased severity with APOE varepsilon4. *Neurochem Int* 2006, *47*, 631-639.
32. Beeri, M.S., Haroutunian, V., Schmeidler, J., Sano, M., Fam, P., Kavanaugh, A., Barr, A.M., Honer, W.G., Katsel, P., Synaptic protein deficits are associated with dementia irrespective of extreme old age. *Neurobiol Aging* 2011, *33*, e1-8.
33. Yi, J.H., Hoover, R., McIntosh, T.K., Hazell, A.S., Early, transient increase in complexin I and complexin II in the cerebral cortex following traumatic brain injury is attenuated by N-acetylcysteine*. J Neurotrauma* 2006, *23,* 86-96.
34. Poon, H.F., Farr, S.A., Banks, W.A., Pierce, W.M., Klein, J.B., Morley, J.E., Butterfield, D.A., Proteomic identification of less oxidized brain proteins in aged senescence-accelerated mice following administration of antisense oligonucleotide directed at the Abeta region of amyloid precursor protein. *Brain Res Mol Brain Res* 2005, *138*, 8-16.
35. Ferrer, I., Gomez, A., Carmona, M., Huesa, G., Porta, S., Riera-Codina, M., Biagioli, M., Gustincich, S., Aso, E., Neuronal hemoglobin is reduced in Alzheimer's disease, argyrophilic grain disease, Parkinson's disease, and dementia with Lewy bodies. *J Alzheimers Dis* 2011, *23*, 537-550.
36. Wu, C.W., Liao, P.C., Yu, L., Wang, S.T., Chen, S.T., Wu, C.M., Kuo, Y.M., Hemoglobin promotes Abeta oligomer formation and localizes in neurons and amyloid deposits. *Neurobiol Dis* 2004, *17*, 367-377.
37. Lee, K.S., Chung, J.H., Lee, K.H., Shin, M.J., Oh, B.H., Hong, C.H., Bioplex analysis of plasma cytokines in Alzheimer's disease and mild cognitive impairment. *Immunol Lett* 2008, *121*, 105-109.
38. Popp, J., Bacher, M., Kölsch, H., Noelker, C., Deuster, O., Dodel, R., Jessen, F., Macrophage migration inhibitory factor in mild cognitive impairment and Alzheimer's disease. *J Psychiatr Res* 2009, *43*, 749-753.
39. Craig-Schapiro, R., Kuhn, M., Xiong, C., Pickering, E.H., Liu, J., Misko, T.P., Perrin, R.J., Bales, K.R., Soares, H., Fagan, A.M., Holtzman, D.M., Multiplexed immunoassay panel identifies novel CSF biomarkers for Alzheimer's disease diagnosis and prognosis. *PLoS One* 2011, *6*, e18850.
40. Bacher, M., Deuster, O., Aljabari, B., Egensperger, R., Neff, F., Jessen, F., Popp, J., Noelker, C., Reese, J.P., Al-Abed, Y., Dodel, R., The role of macrophage migration inhibitory factor in Alzheimer's disease. *Mol Med* 2010, *16*, 116-121.
41. Lasagna-Reeves, C.A., Castillo-Carranza, D.L., Sengupta, U., Clos, A.L., Jackson, G.R., Kayed, R., Tau oligomers impair memory and induce synaptic and mitochondrial dysfunction in wild-type mice. *Mol Neurodegener* 2011, *6*, 39.
42. Gozal, Y.M., Seyfried, N.T., Gearing, M., Glass, J.D., Heilman, C.J., Wuu, J., Duong, D.M., Cheng, D., Xia, Q., Rees, H.D., Fritz, J.J., Cooper, D.S., Peng, J., Levey, A.I., Lah, J.J., Aberrant septin 11 is associated with sporadic frontotemporal lobar degeneration. *Mol Neurodegener* 2011, *6*, 82.
43. Slemmon, J.R., Hughes, C.M., Campbell, G.A., Flood, D.GIncreased levels of hemoglobin-derived and other peptides in Alzheimer's disease cerebellum. *J Neurosci* 1994, *14*, 2225-2235.
44. Canobbio, I., Catricala, S., Balduini, C., Torti, M., Calmodulin regulates the non-amyloidogenic metabolism of amyloid precursor protein in platelets. *Biochim Biophys Acta* 2011, 1813, 500-506.
45. Xie, C.W., Calcium-regulated signaling pathways: role in amyloid beta-induced synaptic dysfunction. *Neuromolecular Med* 2004, 6, 53-64.
