# Supplementary material for: Sporadic Creutzfeldt–Jakob disease subtype-specific alterations of the brain proteome: Impact on Rab3a recycling
Source: Proteomics. 2012 Dec 12;12(23-24):3610–20. doi: 10.1002/pmic.201200201 (PMC3565451; doi:10.1002/pmic.201200201)
Supplement: Supplementary file 3 [file pmic0012-3610-SD3.doc]

| **Nr** | **Protein name** | **UniProt Accession** | **MW**  **[kDa]** | **pI** | **Score** | **Queries matched** | **Sequence coverage**  **[%]** | **MS/MS analysis** |
| --- | --- | --- | --- | --- | --- | --- | --- | --- |
| 1 | **Heat shock protein HSP 90 alpha** | P07900 | 85 | 4.9 | 327 | 19 | 22 | Start - End Observed Mr(expt) Mr(calc) Delta Miss Sequence  47 - 60 520.9598 1559.8576 1559.8155 0.0421 1 R.ELISNSSDALDKIR.Y  61 - 69 520.2668 1038.5190 1038.4869 0.0321 0 R.YESLTDPSK.L  75 - 84 595.8752 1189.7358 1189.6819 0.0540 0 K.ELHINLIPNK.Q  88 - 100 683.3869 1364.7592 1364.7221 0.0371 0 R.TLTIVDTGIGMTK.A Oxidation (M)  101 - 112 621.8849 1241.7552 1241.6979 0.0573 0 K.ADLINNLGTIAK.S  192 - 201 656.3212 1310.6278 1310.5626 0.0652 0 K.EDQTEYLEER.R  284 - 292 576.2958 1150.5770 1150.5506 0.0265 0 K.YIDQEELNK.T  293 - 299 451.2868 900.5590 900.5181 0.0409 0 K.TKPIWTR.N  339 - 345 408.2687 814.5228 814.5065 0.0163 0 R.ALLFVPR.R  346 - 355 632.8470 1263.6794 1263.6360 0.0434 1 R.RAPFDLFENR.K  347 - 355 554.8030 1107.5914 1107.5349 0.0566 0 R.APFDLFENR.K  437 - 443 474.7456 947.4766 947.4389 0.0378 0 K.FYEQFSK.N  447 - 456 584.8135 1167.6124 1167.5632 0.0492 0 K.LGIHEDSQNR.K  447 - 457 432.9069 1295.6989 1295.6582 0.0407 1 K.LGIHEDSQNRK.K  490 - 499 612.8444 1223.6742 1223.6186 0.0556 0 K.HIYYITGETK.D  500 - 510 618.3171 1234.6196 1234.5942 0.0254 0 K.DQVANSAFVER.L  566 - 573 520.2872 1038.5598 1038.5168 0.0430 1 K.TKFENLCK.I Carbamidomethyl (C)  574 - 581 503.3103 1004.6060 1004.5576 0.0485 1 K.IMKDILEK.K Oxidation (M)  621 - 631 610.7659 1219.5172 1219.4849 0.0323 0 R.DNSTMGYMAAK.K 2 Oxidation (M) |
| 2 |  |  |  |  | 320 | 17 | 23 | Start - End Observed Mr(expt) Mr(calc) Delta Miss Sequence  47 - 60 520.9479 1559.8219 1559.8155 0.0064 1 R.ELISNSSDALDKIR.Y  61 - 74 513.9198 1538.7376 1538.7464 -0.0088 1 R.YESLTDPSKLDSGK.E  88 - 100 683.3652 1364.7158 1364.7221 -0.0063 0 R.TLTIVDTGIGMTK.A Oxidation (M)  101 - 112 621.8516 1241.6886 1241.6979 -0.0093 0 K.ADLINNLGTIAK.S  192 - 201 656.2946 1310.5746 1310.5626 0.0120 0 K.EDQTEYLEER.R  284 - 292 576.2873 1150.5600 1150.5506 0.0095 0 K.YIDQEELNK.T  339 - 345 408.2625 814.5104 814.5065 0.0039 0 R.ALLFVPR.R  347 - 355 554.7809 1107.5472 1107.5349 0.0124 0 R.APFDLFENR.K  387 - 400 757.3930 1512.7714 1512.7784 -0.0069 0 R.GVVDSEDLPLNISR.E  437 - 443 474.7303 947.4460 947.4389 0.0072 0 K.FYEQFSK.N  447 - 456 584.7990 1167.5834 1167.5632 0.0202 0 K.LGIHEDSQNR.K  458 - 464 429.7782 857.5418 857.5334 0.0084 1 K.KLSELLR.Y  490 - 499 612.8191 1223.6236 1223.6186 0.0050 0 K.HIYYITGETK.D  500 - 510 618.3044 1234.5942 1234.5942 0.0000 0 K.DQVANSAFVER.L  566 - 573 520.2748 1038.5350 1038.5168 0.0182 1 K.TKFENLCK.I Carbamidomethyl (C)  574 - 581 503.2880 1004.5614 1004.5576 0.0039 1 K.IMKDILEK.K Oxidation (M)  621 - 631 610.7565 1219.4984 1219.4849 0.0135 0 R.DNSTMGYMAAK.K 2 Oxidation (M) |
| 3 |  |  |  |  | 404 | 23 | 22 | Start - End Observed Mr(expt) Mr(calc) Delta Miss Sequence  47 - 58 646.3381 1290.6616 1290.6303 0.0314 0 R.ELISNSSDALDK.I  47 - 60 520.9645 1559.8717 1559.8155 0.0562 1 R.ELISNSSDALDKIR.Y  61 - 69 520.2867 1038.5588 1038.4869 0.0719 0 R.YESLTDPSK.L  75 - 87 530.6458 1588.9156 1588.8685 0.0471 1 K.ELHINLIPNKQDR.T  88 - 100 675.4040 1348.7934 1348.7272 0.0662 0 R.TLTIVDTGIGMTK.A  88 - 100 683.4006 1364.7866 1364.7221 0.0645 0 R.TLTIVDTGIGMTK.A Oxidation (M)  101 - 112 621.8795 1241.7444 1241.6979 0.0465 0 K.ADLINNLGTIAK.S  101 - 112 621.8840 1241.7534 1241.6979 0.0555 0 K.ADLINNLGTIAK.S  174 - 182 482.2291 962.4436 962.4127 0.0309 0 R.TDTGEPMGR.G  284 - 292 576.2862 1150.5578 1150.5506 0.0073 0 K.YIDQEELNK.T  293 - 299 451.2799 900.5452 900.5181 0.0271 0 K.TKPIWTR.N  339 - 345 408.2732 814.5318 814.5065 0.0253 0 R.ALLFVPR.R  346 - 355 632.8497 1263.6848 1263.6360 0.0488 1 R.RAPFDLFENR.K  347 - 355 554.7964 1107.5782 1107.5349 0.0434 0 R.APFDLFENR.K  437 - 443 474.7478 947.4810 947.4389 0.0422 0 K.FYEQFSK.N  447 - 456 584.8143 1167.6140 1167.5632 0.0508 0 K.LGIHEDSQNR.K  447 - 457 432.9084 1295.7034 1295.6582 0.0452 1 K.LGIHEDSQNRK.K  458 - 464 429.7902 857.5658 857.5334 0.0324 1 K.KLSELLR.Y  465 - 483 754.6924 2261.0554 2260.9616 0.0937 1 R.YYTSASGDEMVSLKDYCTR.M  Carbamidomethyl (C); Oxidation (M)  490 - 499 612.8270 1223.6394 1223.6186 0.0208 0 K.HIYYITGETK.D  621 - 631 594.7745 1187.5344 1187.4951 0.0394 0 R.DNSTMGYMAAK.K  621 - 631 602.7833 1203.5520 1203.4900 0.0621 0 R.DNSTMGYMAAK.K Oxidation (M)  621 - 631 610.7745 1219.5344 1219.4849 0.0495 0 R.DNSTMGYMAAK.K 2 Oxidation (M) |
| 4 |  |  |  |  | 101 | 5 | 7 | Start - End Observed Mr(expt) Mr(calc) Delta Miss Sequence  88 - 100 683.3858 1364.7570 1364.7221 0.0349 0 R.TLTIVDTGIGMTK.A Oxidation (M)  101 - 112 621.8685 1241.7224 1241.6979 0.0245 0 K.ADLINNLGTIAK.S  339 - 345 408.2676 814.5206 814.5065 0.0141 0 R.ALLFVPR.R  347 - 355 554.7838 1107.5530 1107.5349 0.0182 0 R.APFDLFENR.K  500 - 510 618.3237 1234.6328 1234.5942 0.0386 0 K.DQVANSAFVER.L |
| 5 | **Aconitate hydratase, mitochondrial** | Q99798 | 85 | 7.4 | 506 | 17 | 23 | Start - End Observed Mr(expt) Mr(calc) Delta Miss Sequence  59 - 68 585.8545 1169.6944 1169.6768 0.0177 0 R.LNRPLTLSEK.I  143 - 160 651.6823 1952.0251 1952.0003 0.0248 1 R.AKDINQEVYNFLATAGAK.Y  145 - 160 877.4633 1752.9120 1752.8682 0.0438 0 K.DINQEVYNFLATAGAK.Y  234 - 245 610.3297 1218.6448 1218.6245 0.0204 0 K.LTGSLSGWSSPK.D  379 - 395 931.4151 1860.8156 1860.7982 0.0174 0 R.VGLIGSCTNSSYEDMGR.S Carbamidomethyl (C);  Oxidation (M)  412 - 424 732.3825 1462.7504 1462.7416 0.0088 0 K.SQFTITPGSEQIR.A  430 - 437 468.2535 9 34.4924 934.4872 0.0052 0 R.DGYAQILR.D  466 - 474 534.2863 1066.5580 1066.5407 0.0173 0 K.NTIVTSYNR.N  507 - 521 562.6060 1684.7962 1684.7944 0.0017 1 K.FNPETDYLTGTDGKK.F  522 - 534 500.9307 1499.7703 1499.7620 0.0083 1 K.FRLEAPDADELPK.G  524 - 534 599.3157 1196.6168 1196.5925 0.0244 0 R.LEAPDADELPK.G  550 - 564 533.9194 1598.7364 1598.7285 0.0079 0 K.DSSGQHVDVSPTSQR.L  565 - 573 551.8238 1101.6330 1101.6070 0.0261 0 R.LQLLEPFDK.W  578 - 587 600.3632 1198.7118 1198.6809 0.0310 0 K.DLEDLQILIK.V  672 - 679 461.7477 921.4808 921.4668 0.0141 0 R.EHAALEPR.H  702 - 717 881.9771 1761.9396 1761.8937 0.0459 0 K.QGLLPLTFADPADYNK.I  731 - 739 501.7927 1001.5708 1001.5546 0.0163 0 K.DFTPGKPLK.C |
| 6 |  |  |  |  | 343 | 17 | 25 | Start - End Observed Mr(expt) Mr(calc) Delta Miss Sequence  59 - 68 585.8633 1169.7120 1169.6768 0.0353 0 R.LNRPLTLSEK.I  69 - 84 614.6522 1840.9348 1840.8955 0.0392 0 K.IVYGHLDDPASQEIER.G  251 - 258 400.7808 799.5470 799.5167 0.0303 0 K.VAGILTVK.G  402 - 409 419.2629 836.5112 836.4868 0.0244 0 K.QALAHGLK.C  412 - 424 732.3925 1462.7704 1462.7416 0.0288 0 K.SQFTITPGSEQIR.A  430 - 437 468.2634 934.5122 934.4872 0.0250 0 R.DGYAQILR.D  466 - 474 534.2943 1066.5740 1066.5407 0.0333 0 K.NTIVTSYNR.N  507 - 521 562.6159 1684.8259 1684.7944 0.0314 1 K.FNPETDYLTGTDGKK.F  522 - 534 500.9427 1499.8063 1499.7620 0.0443 1 K.FRLEAPDADELPK.G  550 - 564 533.9283 1598.7631 1598.7285 0.0346 0 K.DSSGQHVDVSPTSQR.L  578 - 587 600.3711 1198.7276 1198.6809 0.0468 0 K.DLEDLQILIK.V  634 - 648 801.4169 1600.8192 1600.7845 0.0347 0 R.NAVTQEFGPVPDTAR.Y  657 - 671 834.4201 1666.8256 1666.7587 0.0669 0 R.WVVIGDENYGEGSSR.E  672 - 679 461.7585 921.5024 921.4668 0.0357 0 R.EHAALEPR.H  694 - 701 491.8043 981.5940 981.5607 0.0333 1 R.IHETNLKK.Q  702 - 717 881.9894 1761.9642 1761.8937 0.0705 0 K.QGLLPLTFADPADYNK.I  731 - 739 501.7995 1001.5844 1001.5546 0.0299 0 K.DFTPGKPLK.C |
| 7 | **78 kDa glucose-regulated protein** | P11021 | 72 | 5.1 | 92 | 8 | 13 | S tart - End Observed Mr(expt) Mr(calc) Delta Miss Sequence  50 - 60 614.8450 1227.6754 1227.6207 0.0547 0 R.VEIIANDQGNR.I  124 - 138 535.6448 1603.9126 1603.8570 0.0556 0 K.TKPYIQVDIGGGQTK.T  165 - 181 630.0199 1887.0379 1886.9639 0.0740 0 K.VTHAVVTVPAYFNDAQR.Q  186 - 197 617.3463 1232.6780 1232.6183 0.0597 0 K.DAGTIAGLNVMR.I Oxidation (M)  345 - 353 523.8118 1045.6090 1045.5655 0.0435 1 K.VLEDSDLKK.S  440 - 447 443.7887 885.5628 885.5284 0.0345 1 R.NTVVPTKK.S  524 - 532 537.8024 1073.5902 1073.5465 0.0437 0 K.ITITNDQNR.L  533 - 540 493.7851 985.5556 985.5080 0.0477 0 R.LTPEEIER.M |
| 8 |  |  |  |  | 287 | 19 | 31 | S tart - End Observed Mr(expt) Mr(calc) Delta Miss Sequence  47 - 60 519.2675 1554.7807 1554.7862 -0.0056 1 K.NGRVEIIANDQGNR.I  50 - 60 614.8222 1227.6298 1227.6207 0.0091 0 R.VEIIANDQGNR.I  82 - 96 839.4217 1676.8288 1676.8006 0.0283 0 K.NQLTSNPENTVFDAK.R  124 - 138 535.6247 1603.8523 1603.8570 -0.0047 0 K.TKPYIQVDIGGGQTK.T  139 - 152 768.9088 1535.8030 1535.7905 0.0125 0 K.TFAPEEISAMVLTK.M  139 - 152 776.9064 1551.7982 1551.7854 0.0128 0 K.TFAPEEISAMVLTK.M Oxidation (M)  165 - 181 629.9958 1886.9656 1886.9639 0.0017 0 K.VTHAVVTVPAYFNDAQR.Q  186 - 197 617.3170 1232.6194 1232.6183 0.0011 0 K.DAGTIAGLNVMR.I Oxidation (M)  198 - 213 830.4576 1658.9006 1658.8879 0.0128 0 R.IINEPTAAAIAYGLDK.R  325 - 336 510.2513 1527.7321 1527.7391 -0.0071 1 R.AKFEELNMDLFR.S Oxidation (M)  345 - 352 459.7487 917.4828 917.4706 0.0123 0 K.VLEDSDLK.K  345 - 353 523.7923 1045.5700 1045.5655 0.0045 1 K.VLEDSDLKK.S  440 - 447 443.7774 885.5402 885.5284 0.0119 1 R.NTVVPTKK.S  448 - 464 918.9788 1835.9430 1835.9265 0.0165 0 K.SQIFSTASDNQPTVTIK.V  475 - 492 645.3478 1933.0216 1933.0058 0.0158 0 K.DNHLLGTFDLTGIPPAPR.G  524 - 532 537.7888 1073.5630 1073.5465 0.0165 0 K.ITITNDQNR.L  533 - 540 493.7681 985.5216 985.5080 0.0137 0 R.LTPEEIER.M  563 - 573 658.8206 1315.6266 1315.6295 -0.0029 0 R.NELESYAYSLK.N  622 - 633 699.4025 1396.7904 1396.7813 0.0091 0 K.ELEEIVQPIISK.L |
| 9 | **Heat shock cognate 71 kDa protein** | P11142 | 71 | 5.4 | 137 | 13 | 20 | S tart - End Observed Mr(expt) Mr(calc) Delta Miss Sequence  26 - 36 614.8625 1227.7104 1227.6207 0.0897 0 K.VEIIANDQGNR.T  78 - 88 635.8342 1269.6538 1269.5547 0.0991 0 R.FDDAVVQSDMK.H Oxidation (M)  103 - 112 590.8571 1179.6996 1179.6135 0.0861 1 K.VQVEYKGETK.S  113 - 126 816.9537 1631.8928 1631.7753 0.1176 0 K.SFYPEEVSSMVLTK.M Oxidation (M)  129 - 137 497.3006 992.5866 992.5178 0.0688 0 K.EIAEAYLGK.T  160 - 171 600.3885 1198.7624 1198.6670 0.0955 0 K.DAGTIAGLNVLR.I  221 - 236 564.6155 1690.8247 1690.7183 0.1063 0 K.STAGDTHLGGEDFDNR.M  302 - 311 627.3549 1252.6952 1252.6088 0.0865 0 R.FEELNADLFR.G  312 - 319 429.7640 857.5134 857.4494 0.0640 0 R.GTLDPVEK.A  349 - 357 541.3275 1080.6404 1080.5604 0.0801 0 K.LLQDFFNGK.E  501 - 507 402.7558 803.4970 803.4389 0.0582 0 K.ITITNDK.G  510 - 517 495.3035 988.5924 988.5189 0.0736 1 R.LSKEDIER.M  602 - 609 472.7978 943.5810 943.5161 0.0650 0 K.VCNPIITK.L Carbamidomethyl (C) |
| 10 | **Transketolase** | P29401 | 16 | 7.6 | 295 | 12 | 16 | S tart - End Observed Mr(expt) Mr(calc) Delta Miss Sequence  12 - 21 565.3236 1128.6326 1128.6251 0.0076 1 K.LQALKDTANR.L  103 - 114 422.2186 1263.6340 1263.6459 -0.0119 0 K.ISSDLDGHPVPK.Q  233 - 241 489.7939 977.5732 977.5658 0.0075 0 K.HQPTAIIAK.T  303 - 310 469.7378 937.4610 937.4579 0.0032 0 R.MPSLPSYK.V Oxidation (M)  311 - 318 430.2560 858.4974 858.4923 0.0051 1 K.VGDKIATR.K  319 - 327 475.2782 948.5418 948.5392 0.0026 1 R.KAYGQALAK.L  335 - 343 473.2677 944.5208 944.5179 0.0030 0 R.IIALDGDTK.N  344 - 353 600.8187 1199.6228 1199.6186 0.0042 1 K.NSTFSEIFKK.E  457 - 465 458.7561 915.4976 915.5025 -0.0049 0 K.AVELAANTK.G  543 - 550 458.2867 914.5588 914.5549 0.0040 1 R.KLILDSAR.A  595 - 603 471.7886 941.5626 941.5545 0.0081 0 R.SGKPAELLK.M  610 - 617 422.2369 842.4592 842.4610 -0.0018 0 R.DAIAQAVR.G |
| 11 | **Rab GDP dissociation inhibitor alpha** | P31150 | 50 | 5.0 | 766 | 19 | 50 | S tart - End Observed Mr(expt) Mr(calc) Delta Miss Sequence  36 - 54 1074.0600 2146.1054 2146.0106 0.0949 0 R.NPYYGGESSSITPLEELYK.R  36 - 55 768.3992 2302.1758 2302.1117 0.0641 1 R.NPYYGGESSSITPLEELYKR.F  56 - 68 738.8812 1475.7478 1475.7079 0.0400 0 R.FQLLEGPPESMGR.G Oxidation (M)  69 - 79 656.8770 1311.7394 1311.6935 0.0459 1 R.GRDWNVDLIPK.F  80 - 89 568.8278 1135.6410 1135.6060 0.0351 0 K.FLMANGQLVK.M Oxidation (M)  90 - 98 571.3071 1140.5996 1140.5849 0.0148 0 K.MLLYTEVTR.Y Oxidation (M)  104 - 112 514.2677 1026.5208 1026.5386 -0.0178 0 K.VVEGSFVYK.G  119 - 137 696.3487 2086.0243 2085.9598 0.0644 0 K.VPSTETEALASNLMGMFEK.R 2 Oxidation (M)  143 - 156 827.9299 1653.8452 1653.8039 0.0414 0 K.FLVFVANFDENDPK.T  157 - 169 742.8636 1483.7126 1483.6613 0.0513 0 K.TFEGVDPQTTSMR.D Oxidation (M)  211 - 218 469.7411 937.4676 937.4869 -0.0192 0 K.LYSESLAR.Y  222 - 240 1071.1014 2140.1882 2140.0993 0.0889 0 K.SPYLYPLYGLGELPQGFAR.L  279 - 290 738.8702 1475.7258 1475.7079 0.0180 0 K.QLICDPSYIPDR.V Carbamidomethyl (C)  300 - 309 597.3789 1192.7432 1192.7002 0.0431 0 R.IICILSHPIK.N Carbamidomethyl (C)  329 - 348 757.7192 2270.1358 2270.0824 0.0534 1 R.KSDIYVCMISYAHNVAAQGK.Y  Carbamidomethyl (C); Oxidation (M)  330 - 348 715.0211 2142.0415 2141.9874 0.0541 0 K.SDIYVCMISYAHNVAAQGK.Y Carbamidomethyl  (C); Oxidation (M)  365 - 379 861.9965 1721.9784 1721.9087 0.0698 0 K.EVEPALELLEPIDQK.F  424 - 436 517.2365 1548.6877 1548.6701 0.0176 1 R.MAGTAFDFENMKR.K 2 Oxidation (M)  437 - 447 632.8193 1263.6240 1263.5731 0.0509 1 R.KQNDVFGEAEQ.- |
| 12 |  |  |  |  | 554 | 19 | 42 | S tart - End Observed Mr(expt) Mr(calc) Delta Miss Sequence  36 - 55 768.3965 2302.1677 2302.1117 0.0560 1 R.NPYYGGESSSITPLEELYKR.F  56 - 68 730.8756 1459.7366 1459.7129 0.0237 0 R.FQLLEGPPESMGR.G  56 - 68 738.8878 1475.7610 1475.7079 0.0532 0 R.FQLLEGPPESMGR.G Oxidation (M)  69 - 79 656.8647 1311.7148 1311.6935 0.0213 1 R.GRDWNVDLIPK.F  80 - 89 560.8267 1119.6388 1119.6110 0.0278 0 K.FLMANGQLVK.M  80 - 89 568.8259 1135.6372 1135.6060 0.0313 0 K.FLMANGQLVK.M Oxidation (M)  90 - 98 563.3170 1124.6194 1124.5900 0.0295 0 K.MLLYTEVTR.Y  90 - 98 571.3026 1140.5906 1140.5849 0.0058 0 K.MLLYTEVTR.Y Oxidation (M)  104 - 112 514.2529 1026.4912 1026.5386 -0.0474 0 K.VVEGSFVYK.G  143 - 156 827.9352 1653.8558 1653.8039 0.0520 0 K.FLVFVANFDENDPK.T  157 - 169 734.8591 1467.7036 1467.6664 0.0372 0 K.TFEGVDPQTTSMR.D  157 - 169 742.8425 1483.6704 1483.6613 0.0091 0 K.TFEGVDPQTTSMR.D Oxidation (M)  211 - 218 469.7663 937.5180 937.4869 0.0312 0 K.LYSESLAR.Y  279 - 290 738.8513 1475.6880 1475.7079 -0.0198 0 K.QLICDPSYIPDR.V Carbamidomethyl (C)  300 - 309 597.3734 1192.7322 1192.7002 0.0321 0 R.IICILSHPIK.N Carbamidomethyl (C)  329 - 348 757.7184 2270.1334 2270.0824 0.0510 1 R.KSDIYVCMISYAHNVAAQGK.Y  Carbamidomethyl (C); Oxidation (M)  365 - 379 861.9908 1721.9670 1721.9087 0.0584 0 K.EVEPALELLEPIDQK.F  424 - 436 517.2372 1548.6898 1548.6701 0.0197 1 R.MAGTAFDFENMKR.K 2 Oxidation (M)  437 - 447 632.8117 1263.6088 1263.5731 0.0357 1 R.KQNDVFGEAEQ.- |
| 13 |  |  |  |  | 192 | 17 | 34 | S tart - End Observed Mr(expt) Mr(calc) Delta Miss Sequence  29 - 35 457.7688 913.5230 913.4804 0.0427 1 K.KVLHMDR.N Oxidation (M)  56 - 68 730.8972 1459.7798 1459.7129 0.0669 0 R.FQLLEGPPESMGR.G  56 - 68 738.8809 1475.7472 1475.7079 0.0394 0 R.FQLLEGPPESMGR.G Oxidation (M)  80 - 89 568.8218 1135.6290 1135.6060 0.0231 0 K.FLMANGQLVK.M Oxidation (M)  90 - 98 563.3251 1124.6356 1124.5900 0.0457 0 K.MLLYTEVTR.Y  90 - 98 571.2858 1140.5570 1140.5849 -0.0278 0 K.MLLYTEVTR.Y Oxidation (M)  104 - 112 514.2968 1026.5790 1026.5386 0.0404 0 K.VVEGSFVYK.G  143 - 156 827.9410 1653.8674 1653.8039 0.0636 0 K.FLVFVANFDENDPK.T  157 - 169 742.8569 1483.6992 1483.6613 0.0379 0 K.TFEGVDPQTTSMR.D Oxidation (M)  209 - 218 590.3602 1178.7058 1178.6659 0.0400 1 R.IKLYSESLAR.Y  211 - 218 469.7677 937.5208 937.4869 0.0340 0 K.LYSESLAR.Y  211 - 218 469.7744 937.5342 937.4869 0.0474 0 K.LYSESLAR.Y  279 - 290 738.9031 1475.7916 1475.7079 0.0838 0 K.QLICDPSYIPDR.V Carbamidomethyl (C)  300 - 309 597.3799 1192.7452 1192.7002 0.0451 0 R.IICILSHPIK.N Carbamidomethyl (C)  329 - 348 757.7322 2270.1748 2270.0824 0.0924 1 R.KSDIYVCMISYAHNVAAQGK.Y  Carbamidomethyl (C); Oxidation (M)  365 - 379 862.0010 1721.9874 1721.9087 0.0788 0 K.EVEPALELLEPIDQK.F  424 - 436 517.2484 1548.7234 1548.6701 0.0533 1 R.MAGTAFDFENMKR.K 2 Oxidation (M) |
| 14 | **Tubulin beta chain** | P07437 | 50 | 4.8 | 1078 | 14 | 39 | S tart - End Observed Mr(expt) Mr(calc) Delta Miss Sequence  1 - 19 532.2631 2125.0233 2125.0521 -0.0288 1 .MREIVHIQAGQCGNQIGAK.F  Carbamidomethyl (C); Oxidation (M)  3 - 19 608.2944 1821.8614 1821.9156 -0.0542 0 R.EIVHIQAGQCGNQIGAK.F Carbamidomethyl (C)  63 - 77 816.4063 1630.7980 1630.8236 -0.0256 0 R.AILVDLEPGTMDSVR.S Oxidation (M)  155 - 162 539.2672 1076.5198 1076.5250 -0.0052 1 K.IREEYPDR.I  163 - 174 668.3480 1334.6814 1334.6904 -0.0090 0 R.IMNTFSVVPSPK.V Oxidation (M)  242 - 251 565.7738 1129.5330 1129.5880 -0.0549 0 R.FPGQLNADLR.K  242 - 252 420.2290 1257.6652 1257.6830 -0.0178 1 R.FPGQLNADLRK.L  252 - 262 644.3573 1286.7000 1286.7169 -0.0169 1 R.KLAVNMVPFPR.L Oxidation (M) (  263 - 276 546.2778 1635.8116 1635.8232 -0.0116 0 R.LHFFMPGFAPLTSR.G Oxidation (M)  298 - 306 541.2108 1080.4070 1080.4151 -0.0080 0 K.NMMAACDPR.H Carbamidomethyl (C); Oxidation  (M)  298 - 306 549.2141 1096.4136 1096.4100 0.0037 0 K.NMMAACDPR.H Carbamidomethyl (C); 2  Oxidation (M)  310 - 318 520.3032 1038.5918 1038.5862 0.0056 0 R.YLTVAAVFR.G  351 - 359 514.7446 1027.4746 1027.5121 -0.0374 0 K.TAVCDIPPR.G Carbamidomethyl (C)  381 - 390 623.2979 1244.5812 1244.5860 -0.0047 0 R.ISEQFTAMFR.R Oxidation (M) |
| 15a | **Selenium-binding protein 1** | Q13228 | 52 | 5.9 | 133 | 9 | 20 | S tart - End Observed Mr(expt) Mr(calc) Delta Miss Sequence  24 - 34 727.9004 1453.7862 1453.7275 0.0587 0 R.EEIVYLPCIYR.N Carbamidomethyl (C)  53 - 62 429.8922 1286.6548 1286.6190 0.0358 0 K.SPQYCQVIHR.L Carbamidomethyl (C)  94 - 103 542.8638 1083.7130 1083.6652 0.0479 0 K.LVLPSLISSR.I  104 - 114 617.3513 1232.6880 1232.6401 0.0479 0 R.IYVVDVGSEPR.A  246 - 254 513.3246 1024.6346 1024.5917 0.0430 0 K.DGLIPLEIR.F  334 - 344 458.9128 1373.7166 1373.6688 0.0478 0 R.QYDISDPQRPR.L  345 - 357 666.9269 1331.8392 1331.7813 0.0580 0 R.LTGQLFLGGSIVK.G  371 - 379 498.8112 995.6078 995.5651 0.0427 0 K.SQPEPLVVK.G  438 - 448 632.3666 1262.7186 1262.6659 0.0527 0 K.LNPNFLVDFGK.E |
| 15b | **Cytosolic non-specific dipeptidase** | Q96KP4 | 53 | 5.7 | 113 | 11 | 21 | S tart - End Observed Mr(expt) Mr(calc) Delta Miss Sequence  10 - 17 526.7585 1051.5024 1051.4570 0.0454 0 K.YIDENQDR.Y  10 - 20 486.2557 1455.7453 1455.6994 0.0459 1 K.YIDENQDRYIK.K  44 - 53 540.7762 1079.5378 1079.4991 0.0387 0 R.MMEVAAADVK.Q Oxidation (M)  44 - 53 548.7772 1095.5398 1095.4940 0.0458 0 R.MMEVAAADVK.Q 2 Oxidation (M)  69 - 84 844.0301 1686.0456 1685.9716 0.0741 0 K.LPDGSEIPLPPILLGR.L  150 - 159 556.8317 1111.6488 1111.5986 0.0503 0 K.TGQEIPVNVR.F  202 - 211 412.5802 1234.7188 1234.6856 0.0332 1 K.KKPCITYGLR.G Carbamidomethyl (C)  303 - 308 400.7210 799.4274 799.4010 0.0264 0 K.DILMHR.W Oxidation (M)  403 - 413 617.3434 1232.6722 1232.6401 0.0321 0 K.TVFGVEPDLTR.E  431 - 450 700.0255 2097.0547 2096.9797 0.0750 0 K.NVMLLPVGSADDGAHSQNEK.L Oxidation (M) |
| 16 | **Secernin-1** | Q12765 | 46 | 4.7 | 292 | 12 | 25 | S tart - End Observed Mr(expt) Mr(calc) Delta Miss Sequence  17 - 26 517.3210 1032.6274 1032.5968 0.0307 1 R.AKDGLVVFGK.N  19 - 26 417.7462 833.4778 833.4647 0.0131 0 K.DGLVVFGK.N  33 - 51 727.0134 2178.0184 2177.9753 0.0431 0 R.DEVQEVVYFSAADHEPESK.V  52 - 64 790.3893 1578.7640 1578.7712 -0.0071 0 K.VECTYISIDQVPR.T Carbamidomethyl (C)  197 - 205 557.2621 1112.5096 1112.4920 0.0176 0 K.MDAEHPELR.S Oxidation (M)  287 - 302 607.9503 1820.8291 1820.8152 0.0139 0 R.SSPCIHYFTGTPDPSR.S Carbamidomethyl (C)  320 - 332 719.3278 1436.6410 1436.5878 0.0532 0 K.TQSPCFGDDDPAK.K Carbamidomethyl (C)  337 - 343 460.2494 918.4842 918.4559 0.0283 0 R.FQEKPDR.R  356 - 366 623.3217 1244.6288 1244.5997 0.0292 0 R.AIIESDQEQGR.K  356 - 367 458.5815 1372.7227 1372.6946 0.0280 1 R.AIIESDQEQGRK.L  370 - 377 475.7654 949.5162 949.4790 0.0372 0 R.STMLELEK.Q  370 - 377 483.7485 965.4824 965.4739 0.0085 0 R.STMLELEK.Q Oxidation (M) |
| 17 | **Nucleosome assembly protein 1-like 4** | Q99733 | 43 | 4.6 | 134 | 7 | 17 | S tart - End Observed Mr(expt) Mr(calc) Delta Miss Sequence  27 - 36 609.3057 1216.5968 1216.5870 0.0098 0 K.LTDQVMQNPR.V Oxidation (M)  37 - 44 450.2663 898.5180 898.5236 0.0055 0 R.VLAALQER.L  84 - 93 446.2088 1335.6046 1335.6095 0.0049 0 K.FYEEVHDLER.K  95 - 105 664.8539 1327.6932 1327.6812 0.0120 0 K.YAALYQPLFDK.R  139 - 146 445.2762 888.5378 888.5280 0.0098 1 K.SKVVVTEK.A  169 - 186 1076.0547 2150.0948 2150.0453 0.0496 0 R.NVDMLSELVQEYDEPILK.H Oxidation (M)  169 - 186 1076.0564 2150.0982 2150.0453 0.0530 0 R.NVDMLSELVQEYDEPILK.H |
| 18 | **4-trimethylaminobutyraldehyde dehydrogenase** | P49189 | 54 | 5.7 | 128 | 8 | 22 | S tart - End Observed Mr(expt) Mr(calc) Delta Miss Sequence  20 - 30 552.3016 1102.5886 1102.5142 0.0744 0 R.VEPADASGTEK.A  31 - 38 424.7438 847.4730 847.4188 0.0542 0 K.AFEPATGR.V  50 - 59 543.3367 1084.6588 1084.5876 0.0712 0 K.EVNLAVQNAK.A  86 - 101 636.9921 1907.9545 1907.8353 0.1192 1 R.EREDEIATMECINNGK.S Carbamidomethyl (C)  299 - 310 725.4469 1448.8792 1448.7762 0.1030 1 K.EILDKFTEEVVK.Q  317 - 326 564.8356 1127.6566 1127.5822 0.0744 0 K.IGDPLLEDTR.M  412 - 426 770.9482 1539.8818 1539.7682 0.1137 0 R.ANDTTFGLAAGVFTR.D  472 - 481 622.3738 1242.7330 1242.6496 0.0835 0 R.VTIEYYSQLK.T |
| 19 |  |  |  |  | 84 | 4 | 7 | S tart - End Observed Mr(expt) Mr(calc) Delta Miss Sequence  20 - 30 552.2933 1102.5720 1102.5142 0.0578 0 R.VEPADASGTEK.A  31 - 38 424.7369 847.4592 847.4188 0.0404 0 K.AFEPATGR.V  50 - 59 543.3281 1084.6416 1084.5876 0.0540 0 K.EVNLAVQNAK.A  317 - 326 564.8218 1127.6290 1127.5822 0.0468 0 K.IGDPLLEDTR.M |
| 20 | **Phytanoyl-CoA hydroxylase-interacting protein** | Q96FC7 | 42 | 6.0 | 158 | 7 | 17 | S tart - End Observed Mr(expt) Mr(calc) Delta Miss Sequence  23 - 34 716.3830 1430.7514 1430.7187 0.0327 0 K.NLSLEAIQLCDR.D Carbamidomethyl (C)  58 - 67 592.7961 1183.5776 1183.5543 0.0233 0 K.ISNITCDSFK.I Carbamidomethyl (C)  128 - 139 649.3445 1296.6744 1296.6561 0.0183 0 R.TEYTVAVQTASK.Q  164 - 172 540.8358 1079.6570 1079.6339 0.0232 0 K.VHLTQLLEK.A  193 - 199 496.2339 990.4532 990.4447 0.0086 0 K.EYFDYVR.E  250 - 258 563.8039 1125.5932 1125.5818 0.0114 1 R.YRFEIAAEK.L  369 - 376 453.7352 905.4558 905.4389 0.0169 0 K.TCNISVGR.- Carbamidomethyl (C) |
| 21 | **NIF3-like protein** | Q9GZT8 | 42 | 6.2 | 186 | 6 | 18 | S tart - End Observed Mr(expt) Mr(calc) Delta Miss Sequence  144 - 157 494.2796 1479.8170 1479.7616 0.0553 0 K.GLGACTSRPIHPSK.A Carbamidomethyl (C)  158 - 168 419.2167 1254.6283 1254.5741 0.0541 0 K.APNYPTEGNHR.V  169 - 181 792.9217 1583.8288 1583.7468 0.0821 0 R.VEFNVNYTQDLDK.V  188 - 200 648.3653 1294.7160 1294.6517 0.0643 0 K.GIDGVSVTSFSAR.T  209 - 216 495.7863 989.5580 989.4964 0.0616 0 R.INLNCTQK.A Carbamidomethyl (C)  217 - 227 647.8850 1293.7554 1293.6751 0.0803 0 K.ALMQVVDFLSR.N Oxidation (M) |
| 22 | **Guanine nucleotide-binding protein G(o) subunit alpha** | P09471 | 40 | 5.3 | 272 | 15 | 33 | S tart - End Observed Mr(expt) Mr(calc) Delta Miss Sequence  36 - 46 529.2996 1056.5846 1056.6179 -0.0332 0 K.LLLLGAGESGK.S  55 - 67 482.5734 1444.6984 1444.6834 0.0149 0 K.IIHEDGFSGEDVK.Q  87 - 98 664.8201 1327.6256 1327.5966 0.0291 0 R.AMDTLGIEYGDK.E Oxidation (M)  87 - 100 533.2617 1596.7633 1596.7453 0.0179 1 R.AMDTLGIEYGDKER.K  87 - 100 538.5899 1612.7479 1612.7403 0.0076 1 R.AMDTLGIEYGDKER.K Oxidation (M)  106 - 113 483.2342 964.4538 964.4470 0.0068 0 K.MVCDVVSR.M Carbamidomethyl (C)  106 - 113 491.2376 980.4606 980.4420 0.0187 0 K.MVCDVVSR.M Carbamidomethyl (C); Oxidation (M)  131 - 143 791.3797 1580.7448 1580.7042 0.0407 0 R.LWGDSGIQECFNR.S Carbamidomethyl (C)  144 - 154 437.5556 1309.6450 1309.6262 0.0188 1 R.SREYQLNDSAK.Y  146 - 154 534.2579 1066.5012 1066.4931 0.0082 0 R.EYQLNDSAK.Y  155 - 162 522.7434 1043.4722 1043.4924 -0.0201 0 K.YYLDSLDR.I  180 - 193 536.6396 1606.8970 1606.8719 0.0251 1 R.VKTTGIVETHFTFK.N  182 - 193 460.9118 1379.7136 1379.7085 0.0050 0 K.TTGIVETHFTFK.N  199 - 206 446.2283 890.4420 890.4610 -0.0190 0 R.LFDVGGQR.S  272 - 278 418.7380 835.4614 835.4440 0.0175 1 K.KDLFGEK.I |
| 23 | **Alcohol dehydrogenase [NADP+]** | P14550 | 36 | 6.3 | 299 | 12 | 30 | S tart - End Observed Mr(expt) Mr(calc) Delta Miss Sequence  14 - 23 558.3231 1114.6316 1114.6209 0.0108 0 K.MPLIGLGTWK.S  14 - 23 566.3163 1130.6180 1130.6158 0.0022 0 K.MPLIGLGTWK.S Oxidation (M)  35 - 42 464.7433 927.4720 927.4814 -0.0094 0 K.YALSVGYR.H  86 - 96 433.8865 1298.6377 1298.6367 0.0009 0 K.HHPEDVEPALR.K  86 - 97 476.5856 1426.7350 1426.7317 0.0033 1 K.HHPEDVEPALRK.T  128 - 141 548.9026 1643.6860 1643.6886 -0.0026 0 K.NADGTICYDSTHYK.E Carbamidomethyl (C)  146 - 153 407.7600 813.5054 813.4960 0.0095 0 K.ALEALVAK.G  204 - 218 776.3850 1550.7554 1550.7576 -0.0022 0 R.GLEVTAYSPLGSSDR.A  244 - 251 449.2801 896.5456 896.5443 0.0013 0 R.SPAQILLR.W  276 - 287 746.8613 1491.7080 1491.6592 0.0489 0 K.VFDFTFSPEEMK.Q Oxidation (M)  298 - 308 626.3460 1250.6774 1250.6581 0.0194 0 R.YIVPMLTVDGK.R Oxidation (M  298 - 309 469.9291 1406.7655 1406.7592 0.0063 1 R.YIVPMLTVDGKR.V Oxidation (M) |
| 24 | **Malate dehydrogenase, cytoplasmic** | P40925 | 36 | 6.9 | 543 | 14 | 46 | S tart - End Observed Mr(expt) Mr(calc) Delta Miss Sequence  80 - 92 701.3799 1400.7452 1400.7334 0.0119 0 K.DLDVAILVGSMPR.R Oxidation (M)  111 - 121 576.3124 1150.6102 1150.5982 0.0120 1 K.SQGAALDKYAK.K  126 - 142 879.4709 1756.9272 1756.9142 0.0131 0 K.VIVVGNPANTNCLTASK.S Carbamidomethyl (C)  143 - 157 569.6187 1705.8343 1705.8457 -0.0115 1 K.SAPSIPKENFSCLTR.L Carbamidomethyl (C)  150 - 157 513.7321 1025.4496 1025.4600 -0.0104 0 K.ENFSCLTR.L Carbamidomethyl (C)  171 - 179 458.7432 915.4718 915.5025 -0.0307 0 K.LGVTANDVK.N  180 - 199 760.7054 2279.0944 2279.1083 -0.0140 0 K.NVIIWGNHSSTQYPDVNHAK.V  206 - 220 584.6303 1750.8691 1750.8777 -0.0087 1 K.EVGVYEALKDDSWLK.G  221 - 230 582.8100 1163.6054 1163.5935 0.0119 0 K.GEFVTTVQQR.G  239 - 248 505.2713 1008.5280 1008.5273 0.0007 1 R.KLSSAMSAAK.A Oxidation (M)  249 - 255 407.2077 812.4008 812.3963 0.0045 0 K.AICDHVR.D  249 - 255 435.7118 869.4090 869.4178 -0.0087 0 K.AICDHVR.D Carbamidomethyl (C)  299 - 310 697.3531 1392.6916 1392.7038 -0.0121 0 K.FVEGLPINDFSR.E  319 - 334 908.9191 1815.8236 1815.8414 -0.0178 1 K.ELTEEKESAFEFLSSA. |
| 25 | **Ketosamine-3 kinase** | Q9HA64 | 34 | 6.8 | 398 | 13 | 37 | S tart - End Observed Mr(expt) Mr(calc) Delta Miss Sequence  8 - 15 454.2236 906.4326 906.4229 0.0097 0 R.ELGCSSVR.A Carbamidomethyl (C)  16 - 29 448.8760 1343.6062 1343.6001 0.0061 0 R.ATGHSGGGCISQGR.S Carbamidomethyl (C)  51 - 64 786.9022 1571.7898 1571.7575 0.0324 0 R.MFEGEMASLTAILK.T 2 Oxidation (M)  76 - 94 682.6650 2044.9732 2044.9380 0.0351 0 K.VLDAPGGGSVLVMEHMDMR.H 2 Oxidation (M)  76 - 94 687.9901 2060.9485 2060.9330 0.0155 0 K.VLDAPGGGSVLVMEHMDMR.H 3 Oxidation (M)  103 - 115 469.9267 1406.7583 1406.7518 0.0065 0 K.LGAQLADLHLDNK.K  103 - 116 512.6259 1534.8559 1534.8467 0.0092 1 K.LGAQLADLHLDNKK.L  122 - 130 465.7813 929.5480 929.5294 0.0186 1 R.LKEAGTVGR.G  131 - 142 434.8939 1301.6599 1301.6476 0.0122 0 R.GGGQEERPFVAR.F  173 - 182 625.8035 1249.5924 1249.5683 0.0242 0 R.IQPQMDMVEK.E 2 Oxidation (M)  270 - 276 402.7282 803.4418 803.4290 0.0129 1 K.APGFEKR.L  298 - 305 439.2396 876.4646 876.4487 0.0159 0 R.GSSLNIMR.N  298 - 305 447.2332 892.4518 892.4436 0.0082 0 R.GSSLNIMR.N Oxidation (M) |
| 26 | **Carbonyl reductase [NADPH] 1** | P16152 | 30 | 8.5 | 338 | 11 | 36 | S tart - End Observed Mr(expt) Mr(calc) Delta Miss Sequence  16 - 23 399.7825 797.5504 797.5123 0.0381 0 K.GIGLAIVR.D  28 - 38 589.3571 1176.6996 1176.6503 0.0494 0 R.LFSGDVVLTAR.D  113 - 119 421.7772 841.5398 841.4083 0.1316 0 K.TNFFGTR.D  135 - 145 595.8672 1189.7198 1189.6489 0.0710 0 R.VVNVSSIMSVR.A  135 - 145 603.8612 1205.7078 1205.6438 0.0640 0 R.VVNVSSIMSVR.A Oxidation (M)  149 - 157 538.7842 1075.5538 1075.4968 0.0571 0 K.SCSPELQQK.F Carbamidomethyl (C)  160 - 174 846.9711 1691.9276 1691.8287 0.0989 0 R.SETITEEELVGLMNK.F  175 - 181 433.7540 865.4934 865.4545 0.0389 1 K.FVEDTKK.G  187 - 198 641.3416 1280.6686 1280.6037 0.0649 0 K.EGWPSSAYGVTK.I  199 - 206 422.7775 843.5404 843.5178 0.0226 0 K.IGVTVLSR.I  221 - 232 729.9094 1457.8042 1457.7272 0.0771 0 K.ILLNACCPGWVR.T 2 Carbamidomethyl (C) |
| 27 | **14-3-3 protein beta/alpha** | P31946 | 28 | 4.8 | 503 | 12 | 39 | S tart - End Observed Mr(expt) Mr(calc) Delta Miss Sequence  14 - 20 408.7169 815.4192 815.4137 0.0056 0 K.LAEQAER.Y  21 - 29 524.1859 1046.3572 1046.4049 -0.0476 0 R.YDDMAAAMK.A 2 Oxidation (M)  30 - 43 533.5918 1597.7536 1597.7332 0.0204 0 K.AVTEQGHELSNEER.N  44 - 51 454.2328 906.4510 906.5174 -0.0664 0 R.NLLSVAYK.N  63 - 70 452.2641 902.5136 902.5073 0.0064 0 R.VISSIEQK.T  106 - 117 680.8517 1359.6888 1359.7034 -0.0146 0 K.YLIPNATQPESK.V  123 - 129 458.7180 915.4214 915.4273 -0.0058 1 K.MKGDYFR.Y  123 - 129 466.7184 931.4222 931.4222 0.0001 1 K.MKGDYFR.Y Oxidation (M)  130 - 140 591.7908 1181.5670 1181.5564 0.0106 0 R.YLSEVASGDNK.Q  160 - 169 412.8852 1235.6338 1235.6445 -0.0107 1 K.KEMQPTHPIR.L  160 - 169 418.2198 1251.6376 1251.6394 -0.0018 1 K.KEMQPTHPIR.L Oxidation (M)  215 - 224 603.3364 1204.6582 1204.6485 0.0097 0 K.DSTLIMQLLR.D |
| 28 | **14-3-3 protein zeta/delta** | Q6P3U9 | 28 | 4.7 | 684 | 14 | 43 | S tart - End Observed Mr(expt) Mr(calc) Delta Miss Sequence  10 - 18 508.3064 1014.5982 1014.5458 0.0525 1 K.AKLAEQAER.Y  19 - 27 560.7334 1119.4522 1119.4035 0.0488 0 R.YDDMAACMK.S Carbamidomethyl (C); Oxidation  (M)  28 - 41 774.8914 1547.7682 1547.7063 0.0619 0 K.SVTEQGAELSNEER.N  42 - 49 454.2716 906.5286 906.5174 0.0112 0 R.NLLSVAYK.N  61 - 68 445.2739 888.5332 888.4916 0.0416 0 R.VVSSIEQK.T  84 - 91 509.3023 1016.5900 1016.5502 0.0399 1 R.EKIETELR.D  104 - 115 652.8112 1303.6078 1303.6772 -0.0693 0 K.FLIPNASQAESK.V  121 - 127 466.7390 931.4634 931.4222 0.0413 1 K.MKGDYYR.Y  121 - 127 474.7385 947.4624 947.4171 0.0454 1 K.MKGDYYR.Y Oxidation (M)  128 - 138 576.3103 1150.6060 1150.5506 0.0554 0 R.YLAEVAAGDDK.K  128 - 139 427.2339 1278.6799 1278.6456 0.0343 1 R.YLAEVAAGDDKK.G  158 - 167 418.2366 1251.6880 1251.6394 0.0486 1 K.KEMQPTHPIR.L Oxidation (M)  213 - 222 595.3621 1188.7096 1188.6536 0.0560 0 K.DSTLIMQLLR.D  213 - 222 603.3546 1204.6946 1204.6485 0.0461 0 K.DSTLIMQLLR.D Oxidation (M) |
| 29 | **Ubiquitin carboxyl-terminal hydrolase isozyme L1** | P09936 | 25 | 5.3 | 440 | 15 | 60 | S tart - End Observed Mr(expt) Mr(calc) Delta Miss Sequence  1 - 15 605.9785 1814.9137 1814.9092 0.0044 0 -.MQLKPMEINPEMLNK.V  1 - 15 611.3105 1830.9097 1830.9041 0.0055 0 -.MQLKPMEINPEMLNK.V Oxidation (M)  1 - 15 616.6406 1846.9000 1846.8991 0.0009 0 -.MQLKPMEINPEMLNK.V 2 Oxidation (M)  1 - 15 621.9635 1862.8687 1862.8940 -0.0253 0 -.MQLKPMEINPEMLNK.V 3 Oxidation (M)  20 - 27 443.7483 885.4820 885.4821 -0.0000 0 R.LGVAGQWR.F  66 - 78 742.9130 1483.8114 1483.7882 0.0233 1 K.QIEELKGQEVSPK.V  84 - 105 771.0301 2310.0685 2310.1386 -0.0702 0 K.QTIGNSCGTIGLIHAVANNQDK.L  Carbamidomethyl (C)  106 - 115 532.7782 1063.5418 1063.5550 -0.0131 0 K.LGFEDGSVLK.Q  116 - 123 491.2151 980.4156 980.4815 -0.0658 0 K.QFLSETEK.M  136 - 153 656.6220 1966.8442 1966.8915 -0.0473 0 K.NEAIQAAHDAVAQEGQCR.V Carbamidomethyl  (C)  136 - 153 656.6394 1966.8964 1966.8915 0.0049 0 K.NEAIQAAHDAVAQEGQCR.V Carbamidomethyl  179 - 195 620.2972 1857.8698 1857.8931 -0.0233 0 R.MPFPVNHGASSEDTLLK.D Oxidation (M)  179 - 199 743.3752 2227.1038 2227.0943 0.0095 1 R.MPFPVNHGASSEDTLLKDAAK.V  203 - 213 460.5578 1378.6516 1378.6477 0.0039 1 R.EFTEREQGEVR.F  214 - 221 448.2105 894.4064 894.4633 -0.0569 0 R.FSAVALCK.A Carbamidomethyl (C) |
| 30 | **Proteasome subunit beta type-4** | P28070 | 29 | 5.7 | 186 | 5 | 17 | S tart - End Observed Mr(expt) Mr(calc) Delta Miss Sequence  46 - 60 766.4642 1530.9138 1530.8076 0.1062 0 R.TQNPMVTGTSVLGVK.F  132 - 139 492.3108 982.6070 982.5348 0.0722 0 R.AIHSWLTR.A  197 - 211 576.3562 1726.0468 1725.9261 0.1207 1 R.EVLEKQPVLSQTEAR.D  202 - 211 564.8400 1127.6654 1127.5935 0.0720 0 K.QPVLSQTEAR.D  232 - 240 518.8082 1035.6018 1035.5601 0.0418 0 R.FQIATVTEK.G |
| 31 | **Cofilin-1** | Q5E9F7 | 18 | 8.2 | 570 | 8 | 46 | S tart - End Observed Mr(expt) Mr(calc) Delta Miss Sequence  2 - 13 551.8398 1101.6650 1101.6030 0.0621 0 M.ASGVAVSDGVIK.V  35 - 44 591.3270 1180.6394 1180.5798 0.0596 0 K.AVLFCLSEDK.K Carbamidomethyl (C)  35 - 45 437.2496 1308.7270 1308.6748 0.0522 1 K.AVLFCLSEDKK.N Carbamidomethyl (C)  46 - 53 458.2629 914.5112 914.5073 0.0040 0 K.NIILEEGK.E  74 - 81 525.7672 1049.5198 1049.4634 0.0565 1 K.MLPDKDCR.Y Carbamidomethyl (C); Oxidation (M)  82 - 92 669.3405 1336.6664 1336.6187 0.0478 0 R.YALYDATYETK.E  133 - 146 597.6326 1789.8760 1789.8053 0.0707 1 K.HELQANCYEEVKDR.C Carbamidomethyl (C)  133 - 146 597.6355 1789.8847 1789.8053 0.0794 1 K.HELQANCYEEVKDR.C |
| 32 | **Stathmin** | Q93045 | 17 | 5.8 | 177 | 8 | 46 | S tart - End Observed Mr(expt) Mr(calc) Delta Miss Sequence  15 - 27 694.8851 1387.7556 1387.7459 0.0097 0 R.ASGQAFELILSPR.S  44 - 52 537.7916 1073.5686 1073.5604 0.0082 0 K.DLSLEEIQK.K  44 - 53 601.8431 1201.6716 1201.6554 0.0163 1 K.DLSLEEIQKK.L  53 - 60 473.2575 944.5004 944.4927 0.0078 1 K.KLEAAEER.R  63 - 70 456.7451 911.4756 911.4712 0.0044 0 K.SHEAEVLK.Q  77 - 85 570.3090 1138.6034 1138.5982 0.0052 1 R.EHEKEVLQK.A  86 - 95 583.2869 1164.5592 1164.5411 0.0182 0 K.AIEENNNFSK.M  138 - 149 653.7796 1305.5446 1305.5208 0.0238 1 K.ESKDPADETEAD.- |
| 33 | **Destrin** | Q5E9D5 | 18 | 8.1 | 351 | 13 | 56 | S tart - End Observed Mr(expt) Mr(calc) Delta Miss Sequence  2 - 13 645.8096 1289.6046 1289.6034 0.0012 0 M.ASGVQVADEVCR.I Carbamidomethyl (C)  14 - 19 416.7023 831.3900 831.3837 0.0064 0 R.IFYDMK.V Oxidation (M)  22 - 30 546.2788 1090.5430 1090.5328 0.0102 1 R.KCSTPEEIK.K Carbamidomethyl (C)  23 - 31 546.2764 1090.5382 1090.5328 0.0054 1 K.CSTPEEIKK.R Carbamidomethyl (C)  35 - 44 562.3010 1122.5874 1122.5743 0.0131 0 K.AVIFCLSADK.K Carbamidomethyl (C)  35 - 45 626.3482 1250.6818 1250.6693 0.0126 1 K.AVIFCLSADKK.C Carbamidomethyl (C)  46 - 53 474.2473 946.4800 946.4794 0.0007 0 K.CIIVEEGK.E Carbamidomethyl (C)  70 - 78 537.2825 1072.5504 1072.5376 0.0129 0 K.HFVGMLPEK.D Oxidation (M)  70 - 81 502.2386 1503.6940 1503.6963 -0.0023 1 K.HFVGMLPEKDCR.Y Carbamidomethyl (C);  Oxidation (M)  82 - 92 654.3113 1306.6080 1306.6081 -0.0001 0 R.YALYDASFETK.E  115 - 121 400.2028 798.3910 798.3945 -0.0035 0 K.MIYASSK.D  115 - 125 621.8303 1241.6460 1241.6325 0.0135 1 K.MIYASSKDAIK.K Oxidation (M)  133 - 145 513.8918 1538.6536 1538.6532 0.0004 0 K.HECQANGPEDLNR.A |
| 34a | **Small ubiquitin-related modifier 2** | P61956 | 11 | 5.3 | 77 | 2 | 27 | S tart - End Observed Mr(expt) Mr(calc) Delta Miss Sequence  8 - 21 537.6238 1609.8496 1609.8060 0.0436 1 K.EGVKTENNDHINLK.V  22 - 33 617.8348 1233.6550 1233.6354 0.0197 0 K.VAGQDGSVVQFK.I  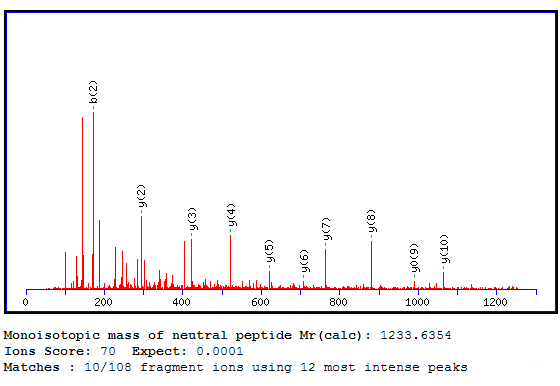 |
| 34b | **Small ubiquitin-related modifier 3** | **P55854** | 11 | 5.3 | 74 | 2 | 24 | S tart - End Observed Mr(expt) Mr(calc) Delta Miss Sequence  8 - 20 499.6086 1495.8040 1495.7630 0.0409 1 K.EGVKTENDHINLK.V  21 - 32 617.8348 1233.6550 1233.6354 0.0197 0 K.VAGQDGSVVQFK.I  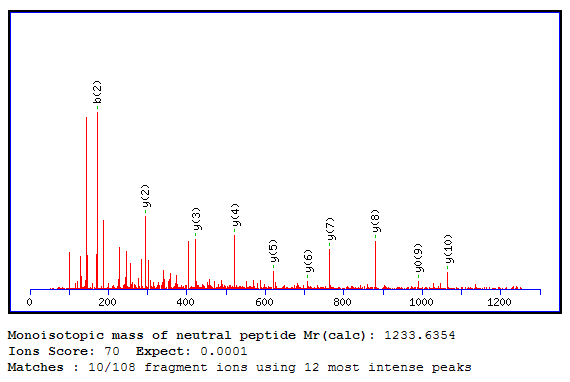 |
| 35a | **Small ubiquitin-related modifier 2** | P61956 | 11 | 5.3 | 77 | 1 | 12 | S tart - End Observed Mr(expt) Mr(calc) Delta Miss Sequence  22 - 33 617.8635 1233.7124 1233.6354 0.0771 0 K.VAGQDGSVVQFK.I  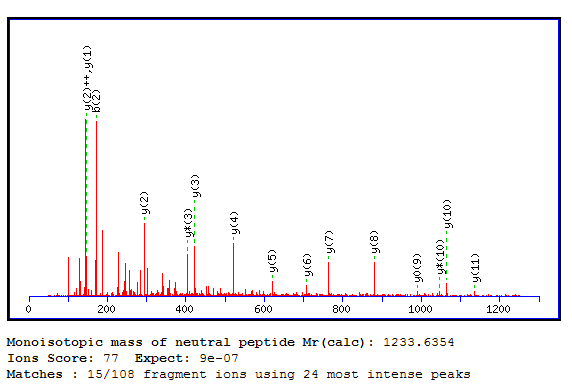 |
| 35b | **Small ubiquitin-related modifier 3** | **P55854** | 11 | 5.3 | 77 | 1 | 12 | S tart - End Observed Mr(expt) Mr(calc) Delta Miss Sequence  22 - 33 617.8635 1233.7124 1233.6354 0.0771 0 K.VAGQDGSVVQFK.  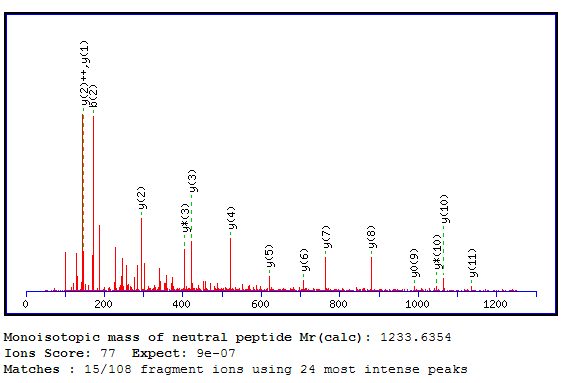 |
| 36 | **Complexin-1** | O14810 | 15 | 4.9 | 172 | 9 | 41 | S tart - End Observed Mr(expt) Mr(calc) Delta Miss Sequence  19 - 32 492.5912 1474.7518 1474.6609 0.0908 1 K.MLGGDEEKDPDAAK.K  19 - 32 497.9242 1490.7508 1490.6559 0.0949 1 K.MLGGDEEKDPDAAK.K Oxidation (M)  55 - 63 545.8057 1089.5968 1089.5237 0.0732 1 K.MEAEREAVR.Q  55 - 63 553.8058 1105.5970 1105.5186 0.0785 1 K.MEAEREAVR.Q Oxidation (M)  79 - 98 702.7109 2105.1109 2104.9695 0.1414 0 R.EAEAQAAMEANSEGSLTRPK.K Oxidation (M)  123 - 133 637.8951 1273.7756 1273.6740 0.1016 0 K.YLPGPLQDMLK.K  123 - 133 645.8907 1289.7668 1289.6689 0.0979 0 K.YLPGPLQDMLK.K Oxidation (M)  123 - 134 473.6250 1417.8532 1417.7639 0.0893 1 K.YLPGPLQDMLKK.- Oxidation (M) |
| 37 | **Profilin-2** | P35080 | 15 | 6.5 | 273 | 8 | 42 | S tart - End Observed Mr(expt) Mr(calc) Delta Miss Sequence  57 - 69 677.8990 1353.7834 1353.6929 0.0906 0 R.EGFFTNGLTLGAK.K  76 - 89 830.3969 1658.7792 1658.6916 0.0876 0 R.DSLYVDGDCTMDIR.T Carbamidomethyl (C)  76 - 89 838.4170 1674.8194 1674.6866 0.1329 0 R.DSLYVDGDCTMDIR.T Carbamidomethyl (C);  Oxidation (M)  92 - 105 717.8618 1433.7090 1433.6899 0.0191 0 K.SQGGEPTYNVAVGR.A  109 - 116 446.8037 891.5928 891.5252 0.0676 0 R.VLVFVMGK.E  109 - 116 454.8049 907.5952 907.5201 0.0751 0 R.VLVFVMGK.E Oxidation (M)  117 - 126 484.2869 966.5592 966.4883 0.0710 0 K.EGVHGGGLNK.K  117 - 127 548.3373 1094.6600 1094.5833 0.0768 1 K.EGVHGGGLNKK.A |
| 38 | **Hemoglobin subunit beta** | Q549N7 | 16 | 6.8 | 604 | 10 | 75 | S tart - End Observed Mr(expt) Mr(calc) Delta Miss Sequence  2 - 9 476.7515 951.4884 951.5025 -0.0141 0 M.VHLTPEEK.S  10 - 18 466.7472 931.4798 931.5127 -0.0329 0 K.SAVTALWGK.V  19 - 31 657.7845 1313.5544 1313.6575 -0.1031 0 K.VNVDEVGGEALGR.L  32 - 41 637.8909 1273.7672 1273.7183 0.0490 0 R.LLVVYPWTQR.F  42 - 60 1037.9651 2073.9156 2073.9354 -0.0197 0 R.FFESFGDLSTPDAVMGNPK.V Oxidation (M)  67 - 83 599.9941 1796.9605 1796.9785 -0.0180 1 K.KVLGAFSDGLAHLDNLK.G  97 - 105 563.7861 1125.5576 1125.5567 0.0009 0 K.LHVDPENFR.L  122 - 133 689.8340 1377.6534 1377.6929 -0.0394 0 K.EFTPPVQAAYQK.V  134 - 145 575.3496 1148.6846 1148.6666 0.0181 0 K.VVAGVANALAHK.Y  134 - 147 483.9301 1448.7685 1448.7888 -0.0203 1 K.VVAGVANALAHKYH. |
| 39 |  |  |  |  | 464 | 9 | 69 | S tart - End Observed Mr(expt) Mr(calc) Delta Miss Sequence  2 - 9 476.7823 951.5500 951.5025 0.0475 0 M.VHLTPEEK.S  10 - 18 466.7961 931.5776 931.5127 0.0649 0 K.SAVTALWGK.V  19 - 31 657.8802 1313.7458 1313.6575 0.0883 0 K.VNVDEVGGEALGR.L  32 - 41 637.9085 1273.8024 1273.7183 0.0842 0 R.LLVVYPWTQR.F  68 - 83 557.3394 1668.9964 1668.8835 0.1129 0 K.VLGAFSDGLAHLDNLK.G  84 - 96 493.6038 1477.7896 1477.6871 0.1024 0 K.GTFATLSELHCDK.L Carbamidomethyl (C)  97 - 105 563.8304 1125.6462 1125.5567 0.0895 0 K.LHVDPENFR.L  122 - 133 689.9030 1377.7914 1377.6929 0.0986 0 K.EFTPPVQAAYQK.V  134 - 145 575.3727 1148.7308 1148.6666 0.0643 0 K.VVAGVANALAHK.Y |
| 40 | **Hemoglobin subunit alpha** | **P69905** | 15 | 8.7 | 257 | 5 | 39 | S tart - End Observed Mr(expt) Mr(calc) Delta Miss Sequence  2 - 12 586.3353 1170.6560 1170.6608 -0.0048 1 M.VLSPADKTNVK.A  18 - 32 765.3824 1528.7502 1528.7270 0.0233 0 K.VGAHAGEYGAEALER.M  33 - 41 544.2491 1086.4836 1086.5420 -0.0583 0 R.MFLSFPTTK.T Oxidation (M)  92 - 100 544.3180 1086.6214 1086.6186 0.0029 1 K.LRVDPVNFK.L  129 - 140 626.8660 1251.7174 1251.7075 0.0100 0 K.FLASVSTVLTSK.Y |
| 41 |  |  |  |  | 348 | 7 | 50 | S tart - End Observed Mr(expt) Mr(calc) Delta Miss Sequence  2 - 12 586.3377 1170.6608 1170.6608 0.0000 1 M.VLSPADKTNVK.A  18 - 32 765.3855 1528.7564 1528.7270 0.0295 0 K.VGAHAGEYGAEALER.M  33 - 41 536.2874 1070.5602 1070.5471 0.0132 0 R.MFLSFPTTK.T  33 - 41 544.2457 1086.4768 1086.5420 -0.0651 0 R.MFLSFPTTK.T Oxidation (M)  42 - 57 611.9407 1832.8003 1832.8846 -0.0843 0 K.TYFPHFDLSHGSAQVK.G  92 - 100 544.2891 1086.5636 1086.6186 -0.0549 1 K.LRVDPVNFK.L  129 - 140 626.8310 1251.6474 1251.7075 -0.0600 0 K.FLASVSTVLTSK.Y |
| 42 | **Macrophage migration inhibitory factor** | P14174 | 12 | 7.7 | 101 | 3 | 17 | S tart - End Observed Mr(expt) Mr(calc) Delta Miss Sequence  2 - 12 644.3817 1286.7488 1286.6805 0.0683 0 M.PMFIVNTNVPR.A  2 - 12 652.3711 1302.7276 1302.6754 0.0522 0 M.PMFIVNTNVPR.A Oxidation (M)  79 - 87 522.8165 1043.6184 1043.5797 0.0387 0 K.LLCGLLAER.L Carbamidomethyl (C)  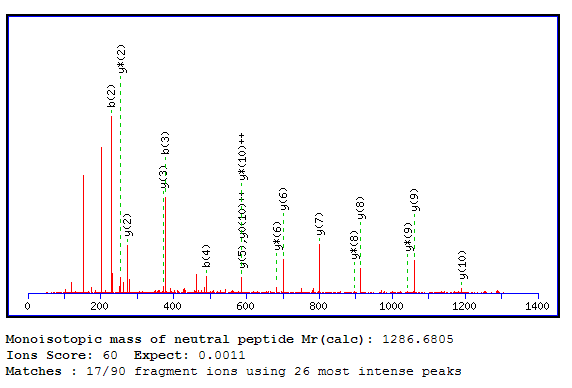 |
| 43 | **Septin-11** | Q9NVA2 | 49 | 6.4 | 292 | 14 | 24 | S tart - End Observed Mr(expt) Mr(calc) Delta Miss Sequence  55 - 65 643.8480 1285.6814 1285.6224 0.0590 0 K.STLMDTLFNTK.F Oxidation (M)  66 - 79 519.2623 1554.7651 1554.7063 0.0588 0 K.FESDPATHNEPGVR.L  84 - 93 612.8200 1223.6254 1223.5782 0.0472 0 R.SYELQESNVR.L  137 - 146 436.9065 1307.6977 1307.6371 0.0606 1 K.RSLFNYHDTR.I  138 - 146 576.8032 1151.5918 1151.5360 0.0559 0 R.SLFNYHDTR.I  163 - 170 453.7699 905.5252 905.4892 0.0361 0 K.SLDLVTMK.K  163 - 170 461.7687 921.5228 921.4841 0.0387 0 K.SLDLVTMK.K Oxidation (M)  163 - 171 525.8234 1049.6322 1049.5791 0.0532 1 K.SLDLVTMKK.L Oxidation (M)  176 - 184 490.8546 979.6946 979.6430 0.0517 0 K.VNIIPIIAK.A  185 - 195 413.9093 1238.7061 1238.6619 0.0442 1 K.ADTIAKNELHK.F  280 - 286 446.7326 891.4506 891.4120 0.0386 0 R.VNMEDLR.E Oxidation (M)  293 - 298 440.7374 879.4602 879.4239 0.0364 0 R.HYELYR.R  327 - 336 617.3659 1232.7172 1232.6513 0.0659 1 K.RNEFLGELQK.K  343 - 348 422.2183 842.4220 842.3779 0.0442 0 R.QMFVMR.V 2 Oxidation (M) |
| 44 | **Beta-centractin** | P42025 | 42 | 5.9 | 282 | 7 | 19 | S tart - End Observed Mr(expt) Mr(calc) Delta Miss Sequence  23 - 32 502.2797 1002.5448 1002.5134 0.0314 0 K.AGFAGDQIPK.Y  47 - 61 767.4230 1532.8314 1532.7909 0.0406 0 R.VMAGALEGDLFIGPK.A Oxidation (M)  73 - 81 552.2880 1102.5614 1102.5230 0.0385 0 R.YPMEHGVVR.D Oxidation (M)  221 - 230 597.3259 1192.6372 1192.5910 0.0462 0 R.ACYLSINPQK.D Carbamidomethyl (C)  239 - 255 894.9874 1787.9602 1787.9054 0.0549 0 K.VQYTLPDGSTLDVGPAR.F  314 - 320 408.7795 815.5444 815.5116 0.0328 1 R.LLSEVKK.L  330 - 336 400.7297 799.4448 799.4188 0.0261 0 K.ISAPQER.L |
| 45 | **Calmodulin** | Q96HK3 | 17 | 4.1 | 214 | 7 | 25 | S tart - End Observed Mr(expt) Mr(calc) Delta Miss Sequence  32 - 38 403.2387 804.4628 804.4164 0.0465 0 K.ELGTVMR.S  32 - 38 411.2360 820.457 4 820.4113 0.0462 0 K.ELGTVMR.S Oxidation (M)  77 - 87 456.8932 1367.6578 1367.5874 0.0703 1 K.MKDTDSEEEIR.E Oxidation (M)  79 - 87 547.2689 1092.5232 1092.4571 0.0662 0 K.DTDSEEEIR.E  79 - 91 532.9366 1595.7880 1595.7063 0.0817 1 K.DTDSEEEIREAFR.V  92 - 107 585.6583 1753.9531 1753.8635 0.0896 1 R.VFDKDGNGYISAAELR.H  96 - 107 633.3522 1264.6898 1264.6048 0.0851 0 K.DGNGYISAAELR.H |
| 46 |  |  |  |  | 305 | 9 | 36 | S tart - End Observed Mr(expt) Mr(calc) Delta Miss Sequence  15 - 22 478.7638 955.5130 955.4651 0.0480 0 K.EAFSLFDK.D  15 - 31 615.6415 1843.9027 1843.8840 0.0187 1 K.EAFSLFDKDGDGTITTK.E  32 - 38 403.2355 804.4564 804.4164 0.0401 0 K.ELGTVMR.S  32 - 38 411.2117 820.4088 820.4113 -0.0024 0 K.ELGTVMR.S Oxidation (M)  77 - 87 456.8855 1367.6347 1367.5874 0.0472 1 K.MKDTDSEEEIR.E Oxidation (M)  79 - 87 547.2567 1092.4988 1092.4571 0.0418 0 K.DTDSEEEIR.E  79 - 91 532.9271 1595.7595 1595.7063 0.0532 1 K.DTDSEEEIREAFR.V  92 - 107 585.6489 1753.9249 1753.8635 0.0614 1 R.VFDKDGNGYISAAELR.H  96 - 107 633.3553 1264.6960 1264.6048 0.0913 0 K.DGNGYISAAELR.H |
